# Lcn2 deficiency leads to long-lasting social impairments independent of maternal immune activation

**DOI:** 10.1186/s12974-026-03742-1

**Published:** 2026-02-25

**Authors:** Martyna Pekala, Sylwia Zawiślak, Sandra Romanis, Karolina Nader, Joanna Dzwonek, Aleksandra Cabaj, Anna Madecka, Alicja Puścian, Ewelina Knapska, Robert Pawlak, Leszek Kaczmarek, Katarzyna Kalita

**Affiliations:** 1 Laboratory of Neurobiology, Nencki-EMBL Partnership for Neural Plasticity and Brain Disorders – BRAINCITY, Nencki Institute of Experimental Biology Polish Academy of Sciences, Warsaw, Poland; 2 Laboratory of Cell Biophysics, Nencki Institute of Experimental Biology Polish Academy of Sciences, Warsaw, Poland; 3 Laboratory of Sequencing, Nencki Institute of Experimental Biology Polish Academy of Sciences, Warsaw, Poland; 4 Laboratory of Emotions Neurobiology, Nencki-EMBL Partnership for Neural Plasticity and Brain Disorders – BRAINCITY, Nencki Institute of Experimental Biology Polish Academy of Sciences, Warsaw, Poland; 5 Laboratory of Neuroeconomics, Centre of New Technologies, University of Warsaw, Warsaw, Poland; 6 Department of Clinical and Biomedical Sciences, University of Exeter Medical School, University of Exeter, Exeter, UK

**Keywords:** Lcn2, maternal immune activation, neurodevelopmental disorders

## Abstract

Maternal infection during pregnancy is a well-established risk factor for neurodevelopmental disorders (NDDs), yet the underlying molecular mechanisms remain poorly understood. Lipocalin-2 (Lcn2), an innate immune protein, is highly upregulated during infection, known to affect neuronal and glial function. This study investigates the role of Lcn2 in shaping brain development, particularly after maternal immune activation (MIA). To mimic maternal infection, pregnant mice received intraperitoneal injections of either lipopolysaccharide (LPS) or saline on embryonic days 16 to 18 to model infection during the second trimester of pregnancy in humans. We first showed that *Lcn2* mRNA was expressed in the fetal brain and that MIA significantly upregulated *Lcn2* mRNA in the hippocampus and neocortex of both sexes. To assess functional relevance, we employed Lcn2 heterozygous females to generate wild-type and Lcn2 KO offspring from the MIA and control groups. Both female and male offspring underwent a battery of behavioral assays. Strikingly, both Lcn2 deletion and MIA independently led to social deficits and increased repetitive behaviors, hallmark features of NDDs, but their combination did not produce additive effects, suggesting a shared or converging mechanism. These alterations were specific to social and repetitive behavior, as no deficits were observed in the learning and memory task. To investigate potential shared molecular mechanisms, we quantified pro-inflammatory cytokine levels in the placenta and fetal forebrain at 4 h after the final LPS injection. No genotype-dependent differences were observed in *IL-6*, *TNF*-α, or *IL-1*β expression. However, RNA sequencing analysis revealed an overlapping group of differentially expressed genes in the Lcn2 KO and MIA groups, indicating convergence on similar transcriptional pathways that may underlie the observed behavioral phenotypes. Together, these findings reveal a previously unrecognized role for Lcn2 in brain development and suggest that while Lcn2 may not directly mediate the damaging effects of maternal immune challenge, it intersects with critical developmental pathways that shape social behavior.

**Supplementary Information:**

The online version contains supplementary material available at 10.1186/s12974-026-03742-1.

## Background

Brain development is a complex and dynamic process that begins early in fetal life and involves the precise formation of neuronal connections essential for cognitive, emotional, and social functioning. A growing body of epidemiological evidence shows that the prenatal period is particularly sensitive to environmental influences, which may disrupt these finely tuned developmental processes and increase the risk of neurodevelopmental disorders (NDDs) [1]. Among these environmental risk factors, prenatal exposure to maternal infections has emerged as a significant contributor, with numerous systematic reviews and meta-analyses linking it to an increased incidence of autism spectrum disorder (ASD), schizophrenia, and attention-deficit/hyperactivity disorder (ADHD) in the offspring [2–7]. Timing appears to be an important factor, with some studies suggesting that infections occurring during the second trimester are particularly associated with a higher risk of NDDs [3, 4].

Although the exact mechanisms by which adverse prenatal exposures lead to NDDs remain unclear, the maternal immune activation (MIA) hypothesis is one of the most prominent explanations. It proposes that the maternal immune response, characterized by elevated levels of circulating cytokines, rather than the pathogen itself, disrupts fetal brain development [8]. Epidemiological studies demonstrate that not only maternal infections, but also chronic inflammatory conditions such as autoimmune diseases, asthma, and obesity, are associated with an increased risk of NDDs in offspring thus supporting the above hypothesis [1]. Further corroborating evidence also comes from animal models, in which maternal immune activation is induced by such agents as bacterial endotoxin lipopolysaccharide (LPS) or synthetic viral RNA analog Poly(I:C), mimicking bacterial and viral infections, respectively [9]. Numerous studies using these models have demonstrated that MIA results in persistent behavioral abnormalities in offspring, accompanied by molecular, anatomical, and functional alterations in the brain [10, 11]. These behavioral impairments often include deficits in social interaction, increased repetitive behaviors, and cognitive dysfunction, hallmarks resembling the core symptoms of various NDDs [11–13]. While such findings strongly support the role of maternal immune signaling in shaping neurodevelopment, the precise molecular mechanisms involved remain poorly defined.

Lipocalin-2 (Lcn2) is an extracellular protein that was initially identified as a part of the innate immune response, released upon infection to bind bacterial siderophores and restrict pathogen growth [14, 15]. Under physiological conditions, Lcn2 levels in the brain are low; however, its expression is markedly upregulated during pathological conditions, including inflammation and following intraperitoneal administration of high doses of LPS [16–23]. Although numerous studies have linked elevated Lcn2 expression to the exacerbation of inflammatory responses, other evidence suggests that Lcn2 may also exert anti-inflammatory effects, highlighting its context-dependent role in immune regulation [21, 24–30].

Importantly, beyond its immunological functions, Lcn2 is essential for maintaining normal neuronal structure and behavior. Lcn2-knockout (Lcn2 KO) mice exhibit anxiety- and depression-like behaviors, along with deficits in spatial learning [31–34]. These behavioral abnormalities are accompanied by impaired adult neurogenesis, altered dendritic arborization, and changes in dendritic spine density. In addition, Lcn2 has been implicated in synaptic plasticity, as in vitro studies show that recombinant Lcn2 reduces membrane expression of NMDA receptor subunits, promotes immature spine morphology, and impairs long-term potentiation (LTP) in hippocampal slices [35, 36].

Despite these insights, the function of Lcn2 in the developing brain, specifically its role in modulating prenatal inflammatory responses, remains unexplored. To address this gap, we have employed a murine MIA model induced by low doses of LPS. We demonstrated that MIA upregulated Lcn2 expression in the fetal brain and induced behavioral abnormalities reminiscent of symptoms associated with NDDs. Strikingly, Lcn2 deletion alone produced comparable behavioral deficits, and combining MIA with Lcn2 deficiency failed to exacerbate these effects, suggesting an occlusion phenomenon. To uncover potential common molecular pathways, we performed bulk RNA sequencing on fetal forebrain tissue and identified a shared set of differentially expressed genes in both Lcn2 KO and MIA groups. These results suggest that, while Lcn2 may not directly mediate the deleterious impact of prenatal immune challenge, it plays a crucial role in normal brain development.

## Materials and methods

### Animals

Wild-type pregnant C57BL/6J and transgenic pregnant females with a knockout of the *Lcn2* gene (strain: B6.129P2-Lcn2tm1Aade/AkiJ, stock #024630, The Jackson Laboratory, USA), maintained on a C57BL/6J genetic background, and Ngal-Luc2/mC (strain: Lcn2tm2Bara Tyrc-2 J/J, #:039157 The Jackson Laboratory, USA) were used in the study [14, 31, 37]. All animals were bred and housed at the Animal Facility of the Nencki Institute under standard laboratory conditions: a 12-h light/dark cycle, ambient temperature maintained at 20–23 °C, relative humidity between 45 and 65%, and 10–15 air changes per hour. Animals had ad libitum access to food and water. Mice were housed in individually ventilated cages (391 × 199 × 160 mm) in groups of 4–5 animals. Pregnant females were separated around gestational day 14 and housed individually until weaning of the offspring. For behavioral testing, males and females were kept in larger cages (395 × 346 × 213 mm) in groups of 9–14 animals. All cages were enriched with poplar wood bedding and nesting materials (wooden blocks, cellulose cotton rolls, and wood wool). All experiments were conducted on offspring from C57BL/6J and Lcn2 Het females. For behavioral studies, 6–8 litters per genotype, per experimental group were used, and for biochemical analyses, 2–4 litters per genotype were included. Behavioral testing was performed in adult offspring, and the same cohort of animals was used across all behavioral assessments. All experimental procedures were performed following the European Communities Council Directive of 24 November 1986 (86/609/EEC), the Animal Protection Act of Poland, and approved by the 1st Local Ethics Committee in Warsaw (approval no. 689/2018, 1169/2021, 1501P1/2023 and 1765/2025).

### Maternal immune activation (MIA) model

A maternal immune activation model was employed to induce systemic immune responses in pregnant mice using intraperitoneal injections of lipopolysaccharide, a component of the outer membrane of Gram-negative bacteria. The MIA protocol was adapted from Schaafsma et al. [38] with modifications. Female mice aged 10–35 weeks were mated with a male overnight. C57BL/6J females were mated with C57BL/6J males, while Lcn2 heterozygous females were mated with either wild-type or knockout males. The following morning, body weight was recorded, and the presence of vaginal plugs was checked to confirm mating. Pregnancy was further verified based on subsequent weight gain. Beginning on gestational day 16, pregnant females received daily intraperitoneal injections of LPS derived from *Escherichia coli* (serotype O111:B4, Sigma-Aldrich, # L4391-1MG) dissolved in sterile saline (Injectio Natrii Chlorati Isotonica, Polpharma, Poland) at a concentration of 5 µg/mL. The dose was 40 µg/kg body weight, administered in a volume appropriate for each animal, for three consecutive days. Control animals received equivalent volumes of sterile saline. Offspring at various developmental stages (prenatal and postnatal) were used for subsequent analyses.

### Sex determination of mouse fetuses by polymerase chain reaction (PCR)

Genomic DNA was isolated from the mice’s tails to determine the sex of the animals. The tissue was incubated in lysis buffer (100 mM NaCl, 100 mM Tris-HCl, 0.5% SDS, 1 mM EDTA, proteinase K 1:10) overnight at 55°C. The next day, proteinase K was inactivated by 10 min incubation at 98°C. The obtained lysate was then diluted in deionized water in a ratio of 1:40 and used as template DNA for amplification using primers 5’-CACCTTAAGAACAAGCCAATACA-3’ and 5’-GGCTTGTCCTGAAAACATTTGG-3 [39].

### Measurement of Lipocalin-2, IL-6, and TNF-α concentration in maternal plasma

Lcn2 levels were quantified in maternal plasma 4 h after the first LPS injection or 24 h after the final LPS injection using the Mouse Lipocalin-2/NGAL DuoSet ELISA kit (# DY1857, R&D Systems, Minneapolis, MN, USA). IL-6 and TNF-α levels were quantified in maternal plasma 4 h after single LPS injection or 4 h after the final LPS injection using, respectively, Mouse IL-6 DuoSet ELISA kit (# DY406, R&D Systems, Minneapolis, MN, USA), Mouse TNF-α DuoSet ELISA kit (# DY410, R&D Systems, Minneapolis, MN, USA), following the manufacturer’s instructions. Blood was collected via cardiac puncture using a 1 ml syringe and transferred to EDTA-coated tubes to prevent coagulation. Samples were centrifuged within 30 min of collection at 4 °C for 15 min at 1000 × g. The plasma fraction was carefully separated and subjected to a second centrifugation at 4 °C for 10 min at 10,000 × g to remove any remaining cellular debris. The plasma was aliquoted and stored at − 80 °C until analysis.

### RNA preparation and quantitative real-time PCR

Total RNA was isolated from the fetal brain using the RNeasy Mini Kit (# 74104, Qiagen, Hilden, Germany). RNA was reverse transcribed with SuperScript™ IV VILO (# 11766050, Thermo Fisher Scientific, Waltham, MA, USA) according to the manufacturer’s instructions. cDNA was amplified with TaqMan probes (*Lcn2* Mm01324470_m1, *Il-1β* Mm00434228_m1, *TNF-α* Mm00443258_m1, *Il-6* Mm00446190_m1, *Gapdh* Mm99999915_g1, ThermoFisher, Waltham, MA, USA) that were specific for the mouse. Quantitative real-time PCR was performed using TaqMan Fast Advanced Master Mix (# 4444556, Thermo Fisher Scientific, Waltham, MA, USA) with StepOne Real-Time PCR System (Thermo Fisher Scientific, MA USA). Fold changes in expression were determined using the ∆∆CT relative quantification method. The values were normalized to relative amounts of Gapdh.

### RNAscope and immunofluorescence

E19 fetuses were anesthetized, then transcardially perfused with PBS, and brains were postfixed in 4% PFA overnight, followed by sucrose. The brain tissues were cryo-sectioned at 15 μm and stored at − 80 °C. Sections were processed according to the manufacturer’s protocol (Bio-Techne USA). Briefly, sections were washed with PBS, baked at 60 °C for 60 min, and fixed in 4% PFA for 15 min at 4 °C. Dehydration was performed with 50%, 70%, and twice with 100% ethanol for 5 min, and sections were incubated with hydrogen peroxide. After washing in distilled water, sections were boiled in 1x target retrieval solution for 5 min, rinsed again in distilled water, and incubated with Protease III for 5 min at 40^◦^C, followed by RNAscope probe (Mm-Lcn2-C1; # 313971, Bio-Techne, USA) at 40^◦^C for 2 h. Signal amplification was achieved through multiple sequential incubations with RNAscope Multiplex Fluorescent Detection Reagents V2 (#323110, Bio-Techne, USA) at 40 °C as follows: AMP1 for 30 min, AMP2 for 30 min, and AMP3 for 15 min. Finally, a signal was developed using channel-specific conjugates of HRP and the Vivid dye (TSA-Vivid 570 for Lcn2). After RNAscope, sections were blocked with 5% normal serum and 0.1% Triton X-100 for 1 h. After overnight incubation at 4 °C with primary antibodies: anti-Laminin (# L9393, Merck), anti-GFAP antibody (# ab4674, Abcam), anti-IBA1 antibody (# 019-19741, FUJIFILM Wako), and anti-NSE (# AB951 Chemicon), the sections were incubated with secondary antibodies and Hoechst 34580 (# H21486, Thermo Fisher Scientific, Waltham, MA, USA). For mCherry immunostaining, brain slices after blocking were incubated with anti-mCherry antibody (# PA5-34974, Thermo Fisher Scientific, Waltham, MA, USA) for 48 h. Next, the sections were incubated with anti-Laminin antibody (# C96142, LSBio, USA), secondary antibodies, and Hoechst. Positive cells were acquired on a Zeiss LSM 800 Airyscan confocal microscope with a PL APO 63x/1.4 oil immersion objective using 488/561/640 nm diode lasers with sequential acquisition settings at 1024 × 1024 resolution, 2.3 × optical zoom, and 0.035 × 0.035 μm pixel size. The settings were kept the same for all scans.

### Western blot analysis

Sixty micrograms of protein extracts collected from the cortex of the E19 fetuses were run on polyacrylamide gels under reducing conditions. The standard Western blot procedure was performed using anti-Lcn2 (# AF 3508, R&D Systems, Minneapolis, MN, USA). To monitor equal total protein levels, the blots were re-probed with β-actin (# A1978, Sigma-Aldrich, Saint Louis, MO, USA) antibodies. The chemiluminescent method was used for signal detection. To quantify individual bands, a scan of X-ray films was analyzed by densitometry using ImageJ.

### Eco-HAB^®^ testing of social behaviors

Eco-HAB^®^ is a fully automated, RFID-based, open-source system designed to assess naturalistic social behaviors in group-housed mice [40]. The apparatus consists of four interconnected polycarbonate compartments (30 × 30 × 18 cm), linked by tubular corridors (internal diameter: 36 mm; external diameter: 40 mm). Mice were automatically tracked by antennas placed on both ends of each corridor based on subcutaneous RFID microchips. The microchips were implanted one week before the experiment under short-term isoflurane anaesthesia. Behavioral data were recorded continuously and analyzed using the Python-based pyEcoHAB library (https://github.com/Neuroinflab/pyEcoHAB).

Mice (8–11 weeks) were tested in the Eco-HAB^®^ system over six consecutive days, following a three-phase protocol: adaptation (days 1–2), sociability assessment (days 3–5), and olfactory stimulus presentation (day 6). At the onset of the dark phase on day 6, soiled bedding from unfamiliar, age- and sex-matched wild-type mice (same strain) was placed behind a perforated partition in one compartment to serve as a social olfactory cue. Clean bedding was used in the opposite compartment as a neutral control.

To characterize spontaneous social interactions within the group, in-cohort sociability was calculated as the total time each pair of mice spent together minus the time they would be expected to spend together based on their individual preferences for occupying specific compartments within the Eco-HAB^®^ system [40, 41]. This parameter was calculated for every pair of animals within each experimental group during dark phases 3, 4, and 5. Additionally, the approach to social odor was evaluated by calculating the proportion of time each animal spent in the compartment containing a novel social odor compared to the time spent in the compartment with a neutral olfactory cue relative to the corresponding period during the preceding dark phase.

### Three-chamber test

The three-chamber test was used to assess social behavior deficits in mice. Testing was conducted in a non-transparent box measuring 63 × 44 × 25 cm, divided into three equal compartments (21 × 44 × 25 cm) by walls containing openings that allowed the animal free access to all chambers. In the center of each of the two outer chambers, an inverted cylindrical metal wire cup (15 cm in height, 12 cm in diameter) was placed to serve as a stimulus container. Mice were tested individually in two consecutive 10-minute phases. During the first phase (habituation), both wire cups were empty, and the mouse was placed in the center chamber with free access to all three compartments to explore the apparatus. In the second phase, one wire cup contained a control olfactory stimulus (clean bedding), while the other contained a social olfactory stimulus (bedding soiled by unfamiliar, age- and sex-matched wild-type conspecifics of the same strain). Behavior was recorded using a video camera connected to a computer and analyzed with EthoVision XT software (Noldus Information Technology, Wageningen, Netherlands), which enables automated tracking of animal movement and interaction. The primary behavioral parameter was the duration of interaction with each wire cup, defined as sniffing, close investigation, or climbing. A social odor preference index (SI) was calculated using the formula: SI = (TS − TNS) / (TS + TNS), where TS represents the time spent interacting with the cup containing the social stimulus, and TNS represents the time spent interacting with the cup containing the control stimulus.

### Marble burying test

The marble burying test was conducted in a clean, transparent plastic cage (27 × 21 × 14 cm) filled with approximately 5 cm of tamped-down bedding to create an even, flat surface. The cage was covered with a second transparent plastic cage of the same dimensions. Twelve identical glass marbles (15 mm in diameter) were arranged in a regular pattern across two rows on the bedding surface. Each mouse was placed individually into the experimental cage and allowed to explore for 15 min. After each session, the mouse was returned to its home cage, and the number of marbles buried, defined as being covered by bedding to at least two-thirds of their depth, was recorded as a measure of repetitive digging behavior.

### Autistic-like score

Autistic-like score is a composite behavioral index designed to quantify autism-like traits, specifically impairments in social interaction, reduced interest in social stimuli, and increased repetitive behaviors. The score was computed based on previously described methods [42, 43], using the standardized values of four behavioral parameters: sociability (EcoHAB^®^ test), approach to social odor (EcoHAB^®^ test), social odor preference index (three-chamber test), and number of marbles buried (marble burying test). For each parameter, standardization was performed as follows: (x – min value) / (max value – min value), where *x* is the raw value for a given animal, and *min* and *max* are the minimum and maximum values across the entire cohort. Given that approach to social odor and social odor preference index assess overlapping behavioral dimensions, their standardized values were averaged so that each of the three behavioral domains (social interaction, social interest, and repetitive behavior) contributed equally to the final score. To ensure consistent directionality of the score (i.e., higher values reflecting stronger autistic-like behavior), the standardized scores for social interaction and social interest were inverted using 1- standardized score. The autistic score was then calculated as: (1- score _social interaction_) + (1 – score _social interest_) + score _repetitive behavior_. This procedure yields a score ranging from 0 to 3, with higher values indicating more pronounced autistic-like traits.

### IntelliCage system for testing reward-motivated learning

IntelliCage (TSE Systems, Berlin, Germany) is an automated system for monitoring behavior in group-housed mice [44, 45]. It consists of a home cage (55 × 37.5 × 20.5 cm) with four experimental corners, each equipped with sensors and controlled via IntelliCagePlus software (v3.3.7.0). Each corner, accessible to one mouse at a time via a 3 cm tube, contains two water bottles behind motorized doors. An overhead sensor detects presence, while an RFID antenna identifies individual mice via implanted transponders, enabling the system to open the doors only for authorized mice following a nosepoke.

An appetitively motivated training paradigm was conducted using the IntelliCage system, enabling mice to acquire a preference for a bottle containing 10% sucrose solution, a highly motivating reward [45, 46]. The procedure began with a two-day adaptation period, with bottle-access doors open and free access to all corners. Next, doors were closed for two days, and mice learned to nosepoke for water access. Doors opened for 5 s following a correct response. At the end of this session, the least preferred corner (i.e., the one with the fewest visits) was identified for each mouse. In the following three-day phase, water access was restricted to the individually least preferred corner. Instrumental responses to both bottles in that corner were recorded. The bottle associated with fewer responses was then replaced with a 10% sucrose solution, available for the next five days. Animal activity, learning to discriminate between bottles containing plain and sucrose-sweetened water, and sucrose preference were assessed.

### RNA Sequencing

Tissue from an E18 mouse forebrain was quickly dissected and stored in RNAlater solution (# AM7020, Invitrogen) at 4 °C for 24 h, supernatant was removed and tissue kept at − 80 °C for further use. Total RNA was isolated using the RNeasy Mini Kit (# 74104, Qiagen, CA, USA) according to the manufacturer’s protocol. Quality and integrity of total RNA were assessed with Agilent 2100 Bioanalyzer using an RNA 6000 Nano Kit (# 5067 − 1511, Agilent Technologies, Ltd., CA, USA) In total, strand-specific polyA-enriched RNA libraries were prepared using the KAPA Stranded mRNA Sample Preparation Kit according to the manufacturer’s protocol (# 07962207001, Kapa Biosystems, MA, USA). Briefly, mRNA molecules were enriched from 500ng of total RNA using poly-T oligo-attached magnetic beads (# 07962207001, Kapa Biosystems, MA, USA). The obtained mRNA was fragmented, and the first-strand cDNA was synthesized using a reverse transcriptase. Second, cDNA synthesis was performed to generate double-stranded cDNA (dsDNA). Adenosines were added to the 3′ ends of dsDNA, and adapters were ligated (# E7600S, adapters from NEB, Ipswich, MA, USA). Following the adapter ligation, uracil in a loop structure of the adapter was digested by USER enzyme from NEB (# E7600S, Ipswich, MA, USA). Adapters containing DNA fragments were amplified by PCR using NEB starters (# E7600S, Ipswich MA, USA). Library evaluation was done with Agilent 2100 Bioanalyzer using the Agilent DNA High Sensitivity chip (# 5067 − 4626, Agilent Technologies, Ltd. CA, USA). The mean library size was 300 bp. Libraries were quantified using a Quantus fluorometer and QuantiFluor double-stranded DNA System (# E4871, Promega, Madison, Wisconsin, USA). Libraries were paired-end sequenced (2 × 151 bp) on NovaSeq 6000 (Illumina, San Diego, CA, USA). RNA sequencing was performed in the Laboratory of Sequencing, Nencki Institute of Experimental Biology, Poland.

### Data preprocessing and analysis

Quality control of reads was performed using FastQC (v. 0.11.9). Adapter and quality trimming were performed using cutadapt (v. 3.4) and TrimGalore (v. 0.6.7), respectively. TrimGalore quality parameter was set to 25. Alignment of reads to the murine reference genome (GRCm39) was performed using STAR (v. 2.7.9a) with default settings. The GTF annotation file was used from the 105 Ensembl release. Duplicate reads were marked using Picard MarkDuplicates (v. 2.27.4-SNAPSHOT). Final quality control was collated with MultiQC (v. 1.13) from RSeQC (v. 3.0.1) and the tools described above. Reads were summarized and counted by featureCounts (v. 2.0.0) on paired-end reads, with only primary alignments and reversely stranded reads counted. The minimum mapping quality score required for a read to be counted was set to 3. The differential analysis was performed using DESeq2 (v. 1.34) with default parameters. If the sum of counts for a specific feature was less than 10 in all samples, this feature was discarded from the analysis. Plots were generated using ComplexHeatmap (v. 2.10.0) packages, GraphPad and overlapping genes were identified using Venn diagram tool http://bioinformatics.psb.ugent.be/webtools/Venn/), followed by the exact hypergeometric probability significance test of the overlap using a web-based program (http://nemates.org/MA/progs/overlap_stats.html) [47]. Significantly differentially expressed genes (DEGs); FDR-adjusted p-value < 0.05 obtained from the RNA sequencing were used as input for the pathway analysis. The functional analysis was performed using Metascape https://www.metascape.org/ [48]. The function of DEGs was investigated using Gene Oncology (GO) of biological processes (BP). For identified DEGs, function and pathway enrichment analysis were carried out using the following ontology sources: Gene Ontology and KEGG Pathway with a *p* < 0.01, a minimum count of 3, and an enrichment factor > 1.5 were collected and grouped into clusters.

### Statistical analyses

Sample sizes and detailed statistical information are provided in the text, figures, or their respective legends. Normality of data distribution was assessed using the D’Agostino-Pearson test. Data with normal distribution were analyzed using Student’s *t*-test, one-way ANOVA, two-way ANOVA, two-way repeated-measures ANOVA, or three-way repeated-measures ANOVA, followed by Šídák’s, Tukey’s, post hoc tests for multiple comparisons as appropriate. For comparisons involving more than two groups with non-normal distributions, logarithmic transformation was applied (Y = Ln (Y), or Y = Ln (Y + 1) for data containing zero values). For two-group comparisons, which do not follow a normal distribution, the non-parametric Mann–Whitney U test was applied. Fetal survival data were analyzed using the chi-square (χ²) test. Differences between experimental groups were considered statistically significant at *p* < 0.05. All analyses were performed using GraphPad Prism (version 8.0.2, GraphPad Software).

## Results

### Temporal expression of Lipocalin-2 in the developing hippocampus and its sensitivity to maternal immune activation

**Fig. 1:**
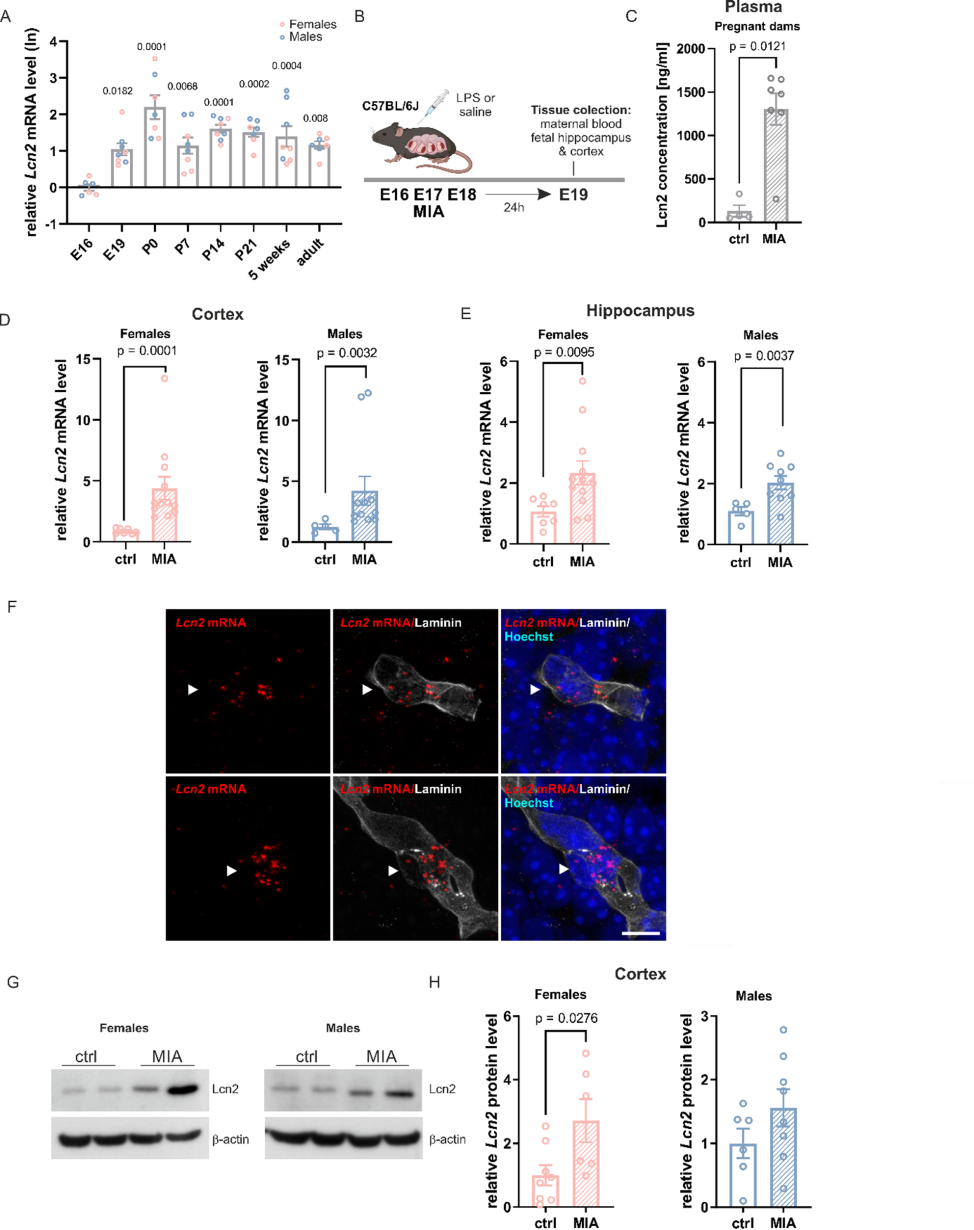
Lipocalin-2 is upregulated in the offspring brain following maternal immune activation induced by LPS. **A** Lcn2 mRNA expression in the hippocampus during pre- and postnatal development in mice. E16 *n* = 6, E19 *n* = 8, P0 *n* = 7, P7 *n* = 8, P14 *n* = 8, P21 *n* = 7, 5 weeks *n* = 8, adulthood *n* = 7 (offspring sex is marked with colors). Data were log-transformed (Y = Ln(Y)) before analysis. One-way ANOVA (F (7, 51) = 8.517, *p* < 0.0001) and Tukey’s post hoc test. **B **Scheme of the experiment. LPS (40 µg/kg) was administered to pregnant C57BL/6J mice on embryonic days E16, E17, and E18. At E19, samples were collected for further analysis. **C** Lcn2 protein level in the plasma from pregnant dams at 24 h after the last LPS injection, measured by enzyme-linked immunosorbent assay (ELISA), ctrl *n* = 4, MIA *n* = 7, Mann-Whitney test analysis. **D**,** E ***Lcn2* mRNA expression levels in the cerebral cortex and hippocampus of female and male fetuses, measured 24 h after the final injection of LPS or saline in pregnant dams, cortex females: ctrl *n* = 7, MIA *n* = 12; males: ctrl *n* = 5, MIA *n* = 11, hippocampus females: ctrl *n* = 7, MIA *n* = 12; males: ctrl *n* = 6, MIA *n* = 10, Mann-Whitney test analysis. **F** Example images of fluorescent *Lcn2* mRNA (RNAscope) and Laminin protein in the cortex at 24 h after MIA. **G** Representative immunoblot of Lcn2 protein in the cortex of female and male offspring 24 h after the last LPS injection. **H** Quantification of immunoblot for Lcn2 band intensity, females: ctrl *n* = 8, MIA *n* = 6; males: ctrl *n* = 6, MIA *n* = 8, Student’s t-test analysis. All data are presented as mean ± SEM, *n* = number of animals

While the role of Lcn2 in the adult brain has been extensively studied [19, 31, 33, 49], its function during brain development remains poorly understood. To determine whether *Lcn2* mRNA in the brain is developmentally regulated, we performed RT-qPCR using RNA isolated from the hippocampi of wild-type C57BL/6J mice of both sexes across various prenatal and postnatal stages, starting from embryonic day 16 (E16) to adult mice (two months old). *Lcn2* mRNA expression was detectable in the hippocampus as early as the prenatal period and remained present at all examined developmental stages (Fig. 1A). The lowest expression levels were observed at E16. A significant increase in Lcn2 expression at consecutive stages of development was observed (E16 vs. E19 *p* = 0.0182; E16 vs. P0 *p* < 0.0001; E16 vs. P7 *p* = 0.0068; E16 vs. P14 *p* < 0.0001; E16 vs. P21 *p* = 0.0002; E16 vs. 5 weeks *p* = 0.0004; E16 vs. adult *p* = 0.0080; Fig. 1A). *Lcn2* mRNA expression peaked at birth (P0), although statistical differences were found only in comparisons with E16 (P0 vs. E16 *p* < 0.0001; Fig. 1A), E19 (P0 vs. E19 *p* = 0.0036; not shown on graph), P7 (P0 vs. P7 *p* = 0.0106; not shown on graph), and adult mice (P0 vs. adult *p* = 0.0177; not shown on graph). During the postnatal period, Lcn2 expression remained relatively stable into adulthood.

To examine how maternal infections affect brain development and whether Lcn2 plays a role in this process, we employed the MIA model. In this approach, pregnant mice were administered bacterial endotoxin to mimic prenatal infection. Beginning on E16, C57BL/6J dams received daily intraperitoneal injections of LPS at a dosage of 40 µg/kg body weight or sterile saline for three consecutive days (Fig. 1B).

To confirm that a low, single dose of LPS (40 µg/kg) induces maternal immune activation, we measured IL-6, TNF-α, and Lcn2 protein levels in plasma from pregnant dams 4 h after injection (Supplementary Fig. 1B, C, D). A significant increase in IL-6 level (control vs. 4 h LPS *p* = 0.0016) and a tendency to increase TNF-α (control vs. 4 h LPS *p* = 0.0606) was observed. A single low dose of LPS also induced a significant increase in the level of Lcn2 protein in the plasma of pregnant dams (control vs. 4 h LPS *p* < 0.0043). Interestingly, repeated LPS injections (MIA model) reduced the expression of IL-6 and TNF-α cytokines, thereby compromising the intended inflammatory response (Supplementary Fig. 1E and F). In contrast, MIA significantly increased Lcn2 levels in the blood 24 h after the last LPS injection (ctrl vs. MIA, *p* = 0.0121, Fig. 1C).

To assess the MIA model’s impact on pregnancy progression, we tracked body weight gain in pregnant females. Pregnant dams were weighed every morning from the day preceding the first injection through the final injection day (E18). We calculated the percent weight gain based on the initial weight recorded on E15. A significant decrease in maternal weight gain was found after the first LPS injection compared to control females receiving saline (after the first dose – E17, ctrl vs. MIA: *p* = 0.0068; Supplementary Fig. 1G). This effect was temporary, as no significant difference was observed after the second injection (after the second dose – E18, ctrl vs. MIA). Additionally, LPS injections notably affected fetal survival, with nearly 50% of pregnancies in the LPS-treated group resulting in stillbirths (χ² test, ctrl vs. MIA: *p* = 0.0058; Supplementary Fig. 1H). There were no differences in the average litter size between groups (Supplementary Fig. 1I). Given that we demonstrated the presence of *Lcn2* mRNA in the developing hippocampus, we aimed to determine whether its expression is sensitive to maternal immune activation. To this end, RT-qPCR was performed using RNA isolated from the cortex and hippocampi of male and female fetuses collected from wild-type C57BL/6J dams 24 h after the final injection of either saline or LPS. Maternal immune activation resulted in a significant upregulation of *Lcn2* mRNA expression in both the cortex (Fig. 1D) and hippocampus (Fig. 1E) of fetuses of both sexes compared to controls (cortex: females, *p* < 0.0001, males, *p* = 0.0032; hippocampus: females, *p* = 0.0095, males, *p* = 0.0037). Notably, the average increase in *Lcn2* mRNA expression was greater in the cortex (females: control 0.87 ± 0.08 vs. MIA 4.39 ± 0.93; males: control 1.26 ± 0.20 vs. MIA 4.22 ± 1.18) than in the hippocampus (females: control 1.07 ± 0.17 vs. MIA 2.34 ± 0.39; males: control 1.09 ± 0.14 vs. MIA 2.03 ± 0.22). To identify the cellular origin of *Lcn2* mRNA, we performed combined RNA fluorescence in situ hybridization with immunofluorescence (RNA-FISH) to co-label *Lcn2* mRNA with different cell markers, including GFAP^+^ astrocytes, NSE^+^ neurons, IBA1^+^ microglia, and Laminin^+^ blood vessels (Fig. 1F and Supplementary Fig. 2A). RNA-FISH revealed that *Lcn2* mRNA is primarily localized to laminin-positive blood vessels after MIA. To confirm this result, we used transgenic animals expressing the Ngal-Luc2/mC reporter, which accurately recapitulate endogenous *Lcn2* mRNA localization [37]. Immunostaining for mCherry revealed a pattern consistent with in situ hybridization, with signal detected in laminin-positive vessels (Supplementary Fig. 2B).

To determine whether Lcn2 was upregulated at the protein level, we performed Western blot analysis on brain samples isolated from fetuses of both sexes. We showed a statistically significant upregulation of Lcn2 protein levels in the female cortex 24 h after the last LPS injection (Fig. 1G, H; *p* = 0.0276). Although a similar increase was observed in males, this result did not reach statistical significance.

### Characterization of MIA responses in Lcn2 heterozygous pregnant females

To assess the potential role of Lcn2 in MIA-induced behavioral changes, we used transgenic animals. Pregnant Lcn2 heterozygous (Lcn2 Het) females received daily intraperitoneal injections of LPS (40 µg/kg) or saline from E16 to E18. Using heterozygous Lcn2 females allowed us to control for maternal genotype-dependent effects on offspring molecular and behavioural outcomes. To assess the potential impact of maternal genotype on the response to LPS, we analyzed cytokine levels in pregnant Lcn2 Het dams. We confirmed that a single dose of LPS increased IL-6 (control vs. 4 h LPS *p* = 0.0114) and TNF-α (control vs. 4 h LPS *p* = 0.0114) levels in maternal plasma at 4 h after injection (Supplementary Fig. 3B, C). No significant differences in cytokine levels were observed between C57BL/6J and Lcn2 Het pregnant dams following a single dose of LPS (Supplementary Fig. 3D, E). To determine how MIA influences pregnancy progression in Lcn2 heterozygotes, we analyzed body weight changes in pregnant females administered either saline or LPS injections. MIA induced by LPS resulted in a significant decrease in maternal weight gain after both the first and second injections compared to the saline-treated controls (E17, control vs. MIA: *p* < 0.0001; E18, control vs. MIA: *p* < 0.0001; Supplementary Fig. 3F). LPS exposure also significantly affected fetal viability, with approximately 50% of pregnancies in the MIA group ending in stillbirths (χ² test, control vs. MIA: *p* = 0.0002; Supplementary Fig. 3G). There were no differences in the average litter size between groups (Supplementary Fig. 3H).

**Fig. 2:**
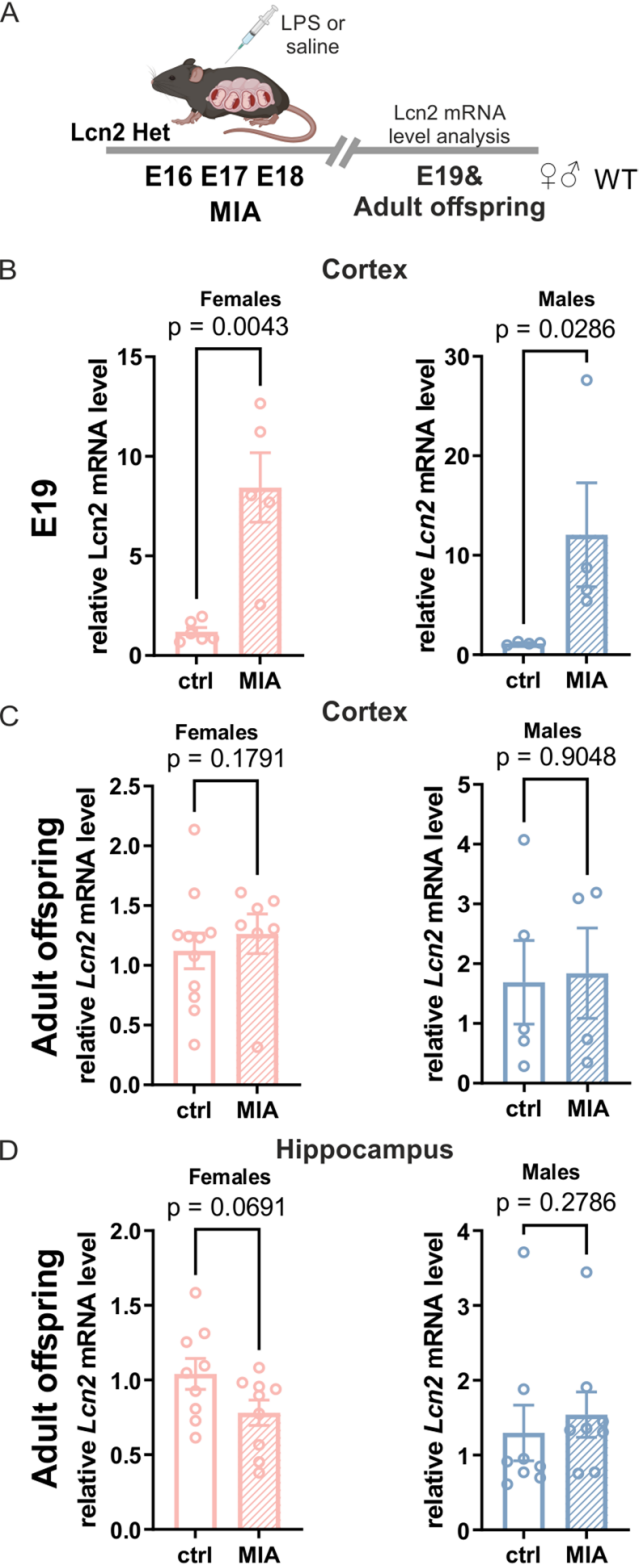
*Lcn2* is upregulated in fetal but not adult brain following MIA. **A** Scheme of the experiment. LPS (40 µg/kg) was administered to pregnant *Lcn2 *Het mice on embryonic days E16, E17, and E18. At E19 and in adulthood, samples were collected for further analysis. **B ***Lcn2 *mRNA expression levels in the cerebral cortex of female and male fetuses, measured 24 h after the final injection of LPS or saline in pregnant dams, cortex females: ctrl *n *= 6, MIA *n *= 5; males: ctrl *n *= 4, MIA *n *= 4, Mann-Whitney test analysis. **C**, **D** Adult cortical and hippocampal *Lcn2 *mRNA expression in female and male offspring following MIA at E16–18; cortex females: ctrl *n *= 11, MIA *n *= 6; males: ctrl *n *= 5, MIA *n *= 4, hippocampus females: ctrl *n* = 9, MIA *n *= 9; males: ctrl *n *= 8, MIA *n *= 8, t test or Mann-Whitney test analysis. All data are presented as mean ± SEM, *n *= number of animals

To confirm that Lcn2 expression is regulated by MIA in Lcn2 Het dams, RT-qPCR was performed using RNA isolated from the cortex of male and female fetuses collected 24 h after the final injection of either saline or LPS. Maternal immune activation resulted in a significant upregulation of *Lcn2* mRNA expression in the cortex (Fig. 2B) of fetuses of both sexes as compared to controls (cortex: females, *p* = 0.0043; males, *p* = 0.0286). To determine whether Lcn2 levels are persistently elevated following MIA, we analyzed *Lcn2* mRNA levels in the adult brain. Interestingly, MIA did not cause a long-term elevation of Lcn2 expression in either the cortex or the hippocampus of adult males or females (Fig. 2C, D). This temporal pattern suggests that Lcn2 primarily shapes early brain development after immune activation, rather than maintaining chronic inflammation.

### Lcn2 deletion mimics behavioral deficits induced by MIA

**Fig. 3:**
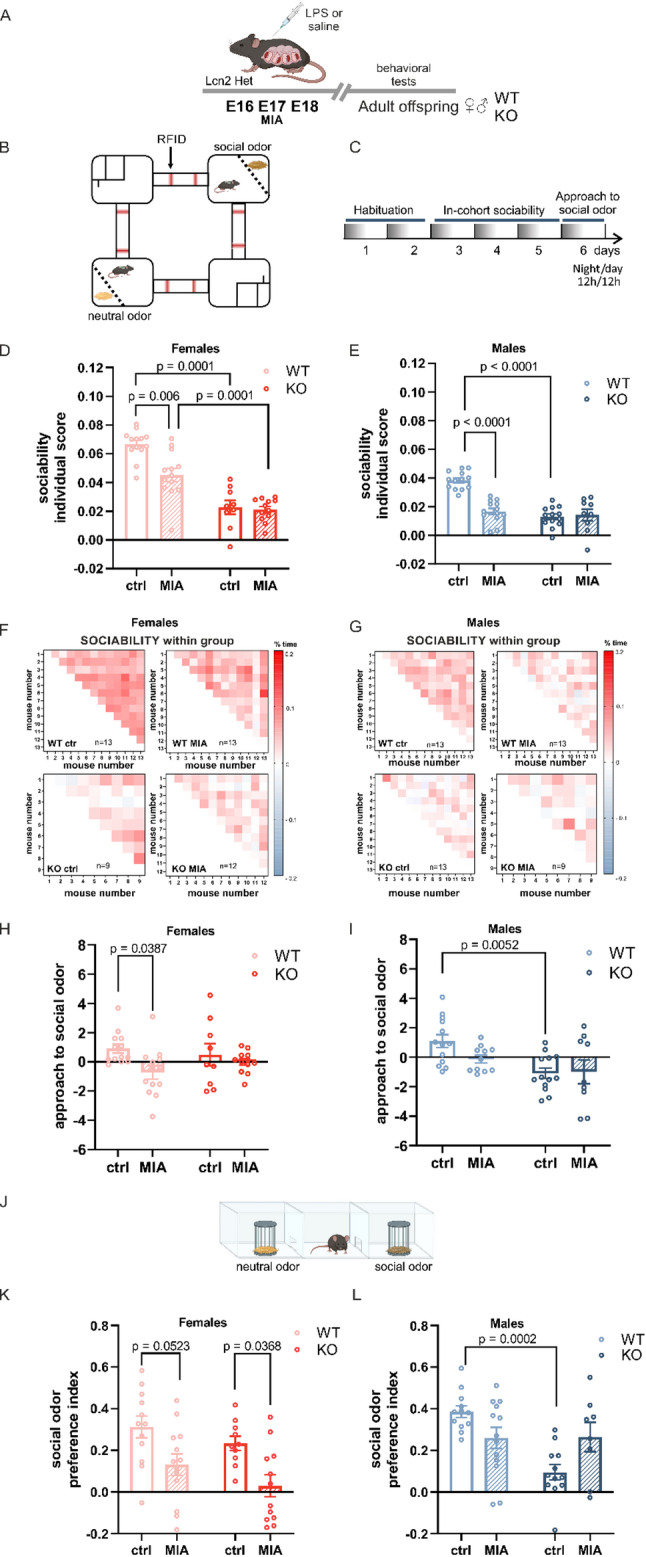
Both maternal immune activation and Lcn2 deletion alter sociability.** A **Scheme of the experiment. LPS was administered to pregnant Lcn2 Het mice on embryonic days E16, E17, and E18. **B** Scheme of the Eco-HAB^®^ apparatus. **C** Timeline of the experiment in the Eco-HAB^®^ system. **D**,** E** Individual score of sociability of females (**D**) and males (**E**) presented as mean value for a given mouse in a group. **D** Two-way ANOVA effects of procedure: F (1,43) = 9.429, *p* = 0.0037, genotype: F (1,43) = 82.52, *p* < 0.0001, and interaction: F (1,43) = 6.795, *p* = 0.0125, followed by Tukey’s post hoc test. **E** Two-way ANOVA effects of procedure: F (1,44) = 18.05, *p* = 0.0001); genotype: F (1,44) = 34.34, *p* < 0.0001; interaction: F (1,44) = 23.09, *p* < 0.0001), followed by Tukey’s post hoc test. **F**,** G** Data from sociability presented as a matrix, where each square represents the time each pair of mice voluntarily spent together. Color intensity corresponds to the strength of their interaction, as indicated by the provided scale. Values from 0 to + 0.2 represent positive social behavior. Values from 0 to -0.2 indicate relative avoidance between that pair. **H**,** I** Approach to social odor of female (**H**) and male (**I**) mice. **H** Two-way ANOVA effect of procedure: F (1, 43) = 5.782; *p* = 0.0206), genotype: F (1, 43) = 5.782; *p* = 0.0206, and interaction: F (1, 43) = 1.666; *p* = 0.2036), followed by Tukey’s post hoc test. **I** Two-way ANOVA effect of procedure: F (1, 43) = 0.3486; *p* = 0.5580, genotype: F (1, 43) = 11.43, *p* = 0.0015, and interaction: F (1, 43) = 0.6216; *p* = 0.4348, followed by Tukey’s post hoc test. Females WT ctrl *n* = 13, WT MIA *n* = 13, KO ctrl *n* = 9, KO MIA *n* = 12; males WT ctrl *n* = 13, WT MIA *n* = 13, KO ctrl *n* = 13, KO MIA *n* = 9. Due to the non-normal distribution of the data, a logarithmic transformation (Y = Ln[Y]) was applied. Data are presented as mean ± SEM. **J** Scheme of the three-chamber social approach task. **K**,** L** Social odor preference index in females (**K**) and males (**L**). **K** Two-way ANOVA effect of procedure: F (1, 43) = 14.88; *p* = 0.0004, genotype: F (1, 43) = 3.273; *p* = 0.0774, and interaction: F (1, 43) = 0.05483, *p* = 0.8160 followed by Tukey’s post hoc test. **L** Two-way ANOVA effect of procedure: F (1, 41) = 0.2154, *p* = 0.6450, genotype: F (1, 41) = 9.547; *p* = 0.0036, and interaction: F (1, 41) = 10.18; *p* = 0.0027, followed by Tukey’s post hoc test. Females: WT ctrl *n* = 12, WT MIA *n* = 13, KO ctrl *n* = 10, KO MIA *n* = 12; males: WT ctrl *n* = 12, WT MIA *n* = 13, KO ctrl *n* = 12, KO MIA *n* = 8. Data are presented as mean ± SEM, *n* = number of animals

After establishing a validated model of MIA characterized by the upregulation of Lcn2 levels in the fetal brain, we examined how maternal infection affects offspring behavior. After weaning, the offspring from various litters were housed together based on treatment, genotype, and sex. Adult Lcn2 KO and wild-type (WT) offspring of both sexes were later evaluated for behaviors associated with NDDs, including social interaction, repetitive behavior, and cognitive function. To assess spontaneous and stimulus-driven social behaviors, we used Eco-HAB^®^, an automated RFID-based system that continuously monitors social behavior in group-housed mice living under seminaturalistic conditions [40, 41]. Behavioral assessments were conducted in adult mice. Eco-HAB^®^ consists of four chambers connected by tube-like corridors; two chambers provide access to food and water, while the other two are equipped with transparent, perforated separators that allow for the presentation of olfactory stimuli without direct physical contact (Fig. 3B). Following a two-day habituation period, when the social structure is variable and still being established [50], social interactions among all pairs of mice were monitored from days 3 to 5 (Fig. 3C). We quantified the in-cohort sociability parameter, which reflects the time mice voluntarily spent with other group members. We found that MIA significantly reduced social interactions in WT offspring of both sexes (Fig. 3D, E, F, G, Supplementary Fig. 4A, E). Notably, deletion of the *Lcn2* gene in saline-treated mice led to a similar decrease in social behaviour, both male and female KO mice exhibited reduced in-cohort sociability compared to WT controls (Fig. 3D, E, F, G, Supplementary Fig. 4B, F). Interestingly, MIA did not exacerbate this deficit in Lcn2 KO mice (Fig. 3D, E, F, G, Supplementary Fig. 4D, H). We also evaluated the approach to social odor, specifically, the preference for novel social cues. This parameter was quantified as the proportion of time spent investigating social (bedding from unfamiliar mice) versus non-social (clean bedding) odors presented behind the perforated separators in the opposing Eco-HAB^®^ compartments on day 6 of the experiment in comparison to the same proportion calculated during a control period, 24 h prior. In WT females, prenatal LPS exposure decreased interest in the social stimulus compared to vehicle-treated controls (WT ctrl vs. WT MIA: *p* = 0.0387, Fig. 3H). A similar trend was noted in males; however, it did not reach statistical significance (*p* = 0.25). Conversely, deletion of the Lcn2 gene diminished social odor preference only in vehicle-treated males (WT ctrl vs. KO control: *p* = 0.0052, Fig. 3I).

To independently validate the findings of Eco-HAB^®^ using a classical and well-established method, we assessed social behavior in the three-chamber test (Fig. 3J). This apparatus evaluates the preference for approaching social and non-social stimuli. To ensure consistency with the Eco-HAB^®^ paradigm, we used clean bedding as the control stimulus, while bedding from unfamiliar mice served as the social stimulus, which was presented under wire cups. We recorded the time the mouse spent interacting with each cup, and a social odor preference index was calculated. Consistent with the Eco-HAB^®^ results, both MIA and *Lcn2* gene deletion reduced the preference for social odor in a sex-dependent manner (Fig. 3K, L). In females, we observed a decreased interest in the social olfactory stimulus following prenatal LPS injections in wild-type mice, with the difference approaching statistical significance (WT ctrl vs. WT MIA: *p* = 0.0523; Fig. 3K). A similar effect of MIA was noted in female knockout mice (KO ctrl vs. KO MIA: *p* = 0.0368; Fig. 3K). In males, the three-chamber results aligned with those from the Eco-HAB^®^ test, demonstrating that social odor preference was reduced exclusively in control males lacking the *Lcn2* gene (WT ctrl vs. KO ctrl: *p* = 0.0002; Fig. 3L).

**Fig. 4:**
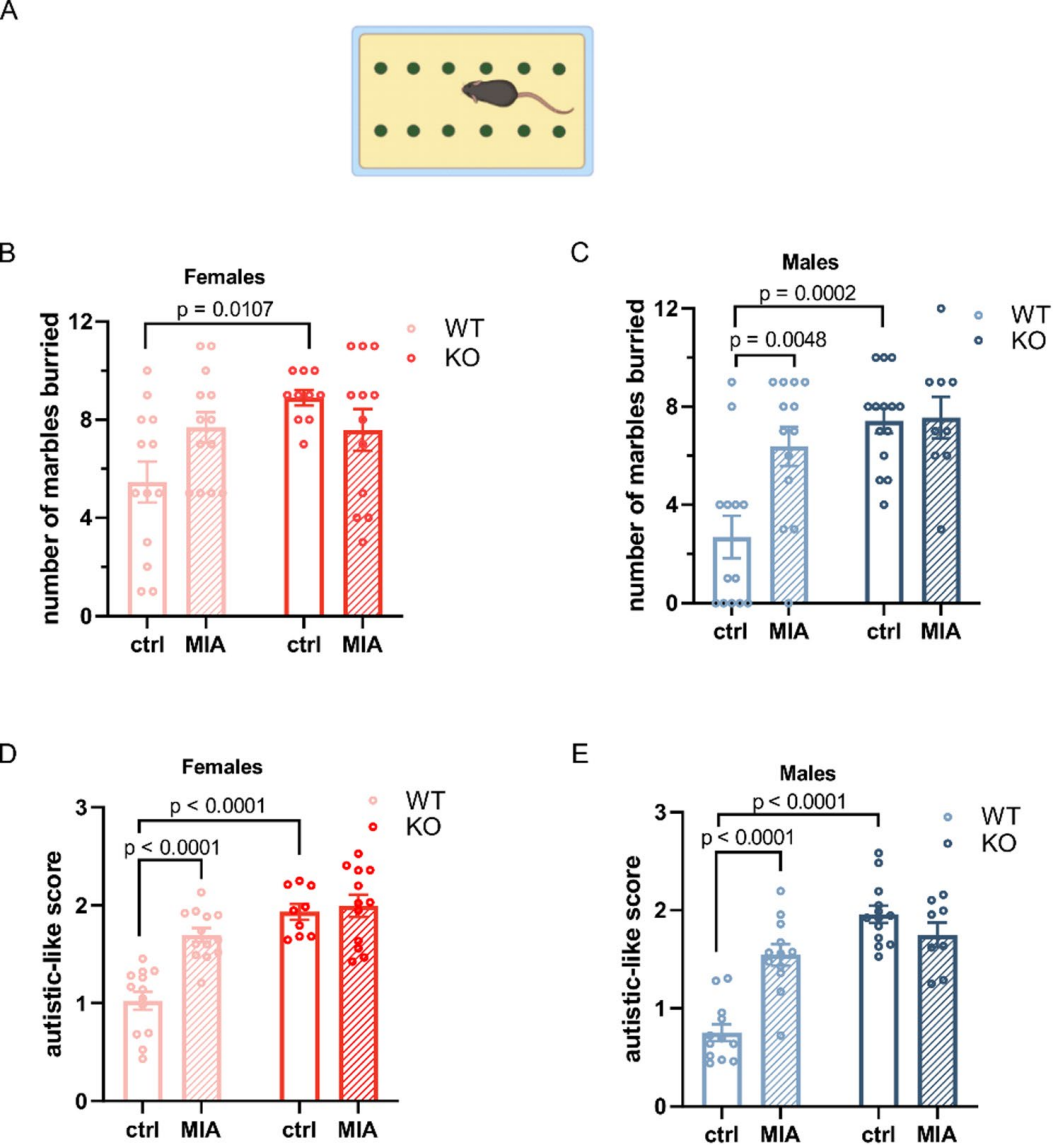
MIA does not exacerbate core autistic-like behaviors in Lcn2 KO mice. **A** Scheme of the marble burying test. **B**,** C** Number of buried marbles in females (**B**) and males (**C**). **B** Two-way ANOVA procedure: F (1, 44) = 0.3982; *p* = 0.5313, genotype: F (1, 44) = 5.283, *p* = 0.0263, and interaction: F (1, 44) = 5.997; *p* = 0,0184, followed by Tukey’s post hoc test. **C** Two-way ANOVA procedure: F (1, 45) = 6.185; *p* = 0.0167, genotype: F (1, 45) = 14.80; *p* = 0.0004, interaction: F (1, 45) = 5.390; *p* = 0.0248, followed by Tukey’s post hoc test. Females: WT ctrl *n* = 13, WT MIA *n* = 13, KO ctrl *n* = 10, KO MIA *n* = 12; males: WT ctrl *n* = 13, WT MIA *n* = 13, KO ctrl *n* = 14, KO MIA *n* = 9. Data are presented as mean ± SEM. **D**,** E** Autistic score: analyzed core symptoms of ASD in females (**D**) and males (**E**). **D** Two-way ANOVA genotype: F (1, 43) = 41.96; *p* < 0.0001, procedure: F (1, 43) = 15.54; *p* = 0.0003, and interaction: F (1, 43) = 10.8; *p* = 0.0020, followed by Tukey’s post hoc test. **E** Two-way ANOVA: genotype: F (1, 41) = 47.73; *p* < 0.0001, procedure: F (1, 41) = 8.257; *p* = 0.0064, and interaction: F (1, 41) = 24.02; *p* < 0.0001, followed by Tukey’s post hoc test. Females: WT ctrl *n* = 13, WT MIA *n* = 13, KO ctrl *n* = 9, KO MIA *n* = 12; males: WT ctrl *n* = 12, WT MIA *n* = 12, KO ctrl *n* = 13, KO MIA *n* = 8. Data are presented as mean ± SEM, *n* = number of animals

Next, we evaluated the impact of maternal immune activation and *Lcn2* gene deletion on repetitive behaviors, one of the hallmark features of ASD, by employing the marble-burying test (Fig. 4A). Mice naturally show digging and burying behaviors; however, an increase in these actions is often utilized as a measure for repetitive behavior [51, 52]. Both female and male control mice lacking the *Lcn2* gene buried significantly more marbles than their wild-type counterparts (WT ctrl vs. KO ctrl: females, *p* = 0.0107, Fig. 4B; males, *p* = 0.0002, Fig. 4C). Furthermore, in males, prenatal LPS exposure resulted in a higher number of buried marbles in wild-type offspring compared to controls (WT ctrl vs. WT MIA: *p* = 0.0048; Fig. 4C), while a similar trend was observed in females, although it did not reach statistical significance (*p* = 0.11; Fig. 4B).

To capture the severity of the core traits associated with autism, specifically, reduced social interaction, a lower tendency for social stimuli, and increased repetitive behavior, we developed a composite behavioral score (from [42] with modification). This method demonstrated a significant increase in autism-related behaviors in WT mice subjected to MIA and in saline-treated Lcn2 KO mice of both sexes, compared to WT control offspring (Fig. 4D, E). Notably, the combination of *Lcn2* gene deletion and prenatal immune activation did not exacerbate the behavioral phenotype. KO MIA mice exhibited similar autistic scores to both WT MIA and KO control groups (Fig. 4D, E), indicating no additive effect. This result suggests that maternal immune activation and Lcn2 deficiency may influence overlapping or converging neurodevelopmental pathways.

### Reward-motivated learning remains unchanged following Lcn2 deletion or maternal immune activation

**Fig. 5:**
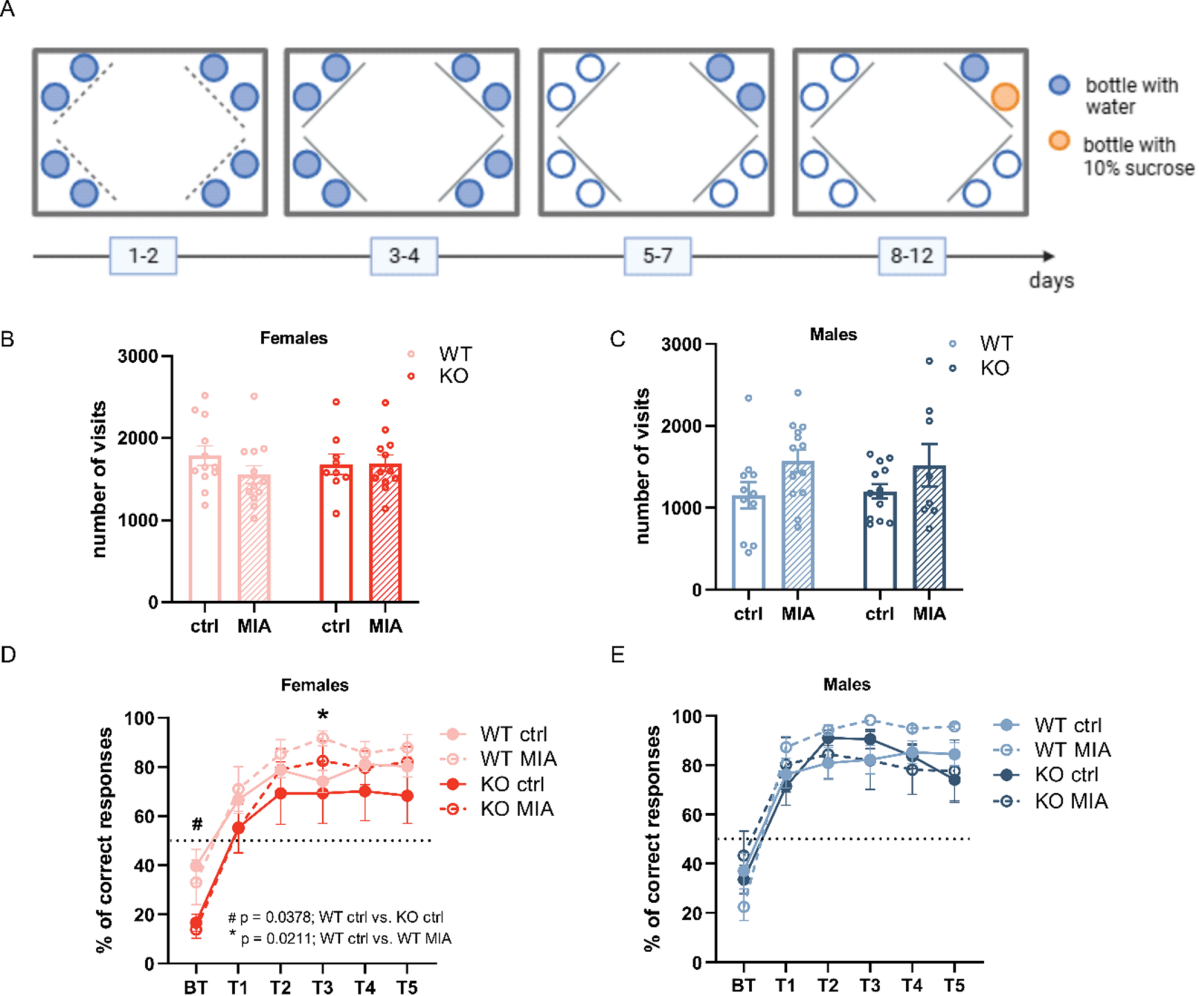
Maternal immune activation and *Lcn2* deletion do not affect appetitive learning in the IntelliCage system. **A** Timeline of the experiment in the IntelliCage. **B**, **C** Activity of females (**B**) and males (**C**) presented as the number of visits to all corners during the experiment. **B **Two-way ANOVA: procedure: F (1, 42) = 0.9213; *p* = 0.3426, genotype F (1, 42) = 0.02222; *p* = 0.8822; and interaction: F (1, 42) = 1.136; *p* = 0.2925; **C** Two-way ANOVA: procedure: F (1, 41) = 5.680; *p* = 0.0219, genotype: F (1, 41) = 0.0001650; *p* = 0.9898; and interaction: F (1, 41) = 0.1022; *p* = 0.7508, followed by Tukey’s post hoc test. **D**, **E** Appetitive learning in females (**D**) and males (**E**) is shown as the percentage of correct responses, defined as visits in which the first nose poke was directed at the door leading to the bottle containing the sugar solution, relative to the total number of visits to that corner in a given session. **D** Three-way ANOVA: session: F (2.448, 102.8) = 89.93; *p* < 0.0001, procedure: F (1, 42) = 1.132, *p* = 0.2935), genotype: F (1, 42) = 3.444, *p* = 0.0705, interaction session x procedure: F (5, 210) = 2.388, *p* = 0.0392, session x genotype: F (5, 210) = 1.417, *p* = 0.2193, procedure x genotype: F (1, 42) = 0.01461, *p* = 0.9044, and session x procedure x genotype: F (5, 210) = 0.2746; *p* = 0.9267 with Tukey’s post hoc: BT: WT ctrl vs. KO ctrl *p* = 0.0378, T3: WT ctrl vs. WT MIA, *p* = 0.0211. **E** Three-way ANOVA: session: F (2,567, 105,2) = 88.58; *p* < 0.0001, procedure: F (1, 41) = 0.6988; *p* = 0.4080, genotype F (1, 41) = 0.7310; *p* = 0.3975, session x procedure: F (5, 205) = 0.9221; *p* = 0.4676, session x genotype: F (5, 205) = 3.163; *p* = 0.0090, procedure x genotype: F (1, 41) = 0.6314; *p* = 0.4314, and session x procedure x genotype: F (5, 205) = 3.925, *p* = 0.0020 followed by Tukey’s post hoc. PT - visits from the three days preceding the appetitive training, when water bottles were present in the corners; T1-T5 - visits from consecutive days of the training sessions. Females - WT ctrl *n* = 12, WT MIA *n* = 13, KO ctrl *n* = 9, KO MIA *n* = 12; males - WT ctrl *n* = 11, WT MIA *n* = 13, KO ctrl *n* = 13, KO MIA *n* = 8. Data are presented as mean ± SEM, *n* = number of animals

Cognitive impairments are a common feature of many NDDs and can be assessed through learning and memory performance [53]. To examine whether Lcn2 gene deletion and maternal immune activation influence cognitive abilities, we employed a reward-motivated learning paradigm using the IntelliCage system. This automated behavioral platform allows for continuous monitoring of voluntary activity in a home-cage-like environment. In this task, mice were trained to discriminate between two drinking bottles in a designated cage corner. One bottle dispensed plain water, while the other provided a palatable sucrose solution as a positive reinforcement. The experimental design is illustrated in Fig. 5A.

Throughout the experiment, we evaluated several behavioral parameters, including general activity, learning of bottle discrimination, and sucrose preference (Fig. 5 and Supplementary Fig. 5). We assessed animal activity by quantifying the total number of corner visits across all experimental phases. No significant differences in overall activity were noted between experimental groups for either female or male mice (Fig. 5B, C).

Appetitive learning was assessed by calculating the percentage of visits where the first nose poke was directed at the door leading to the sucrose-containing bottle [45]. This measure was analyzed across consecutive training sessions to evaluate the acquisition of a preference for the sweetened reward. Data from the three days preceding the appetitive training (before training, BT; averaged) and the five subsequent training sessions (T1-T5) are shown (Fig. 5D, E). All experimental groups of female and male mice exhibited a significant increase in the percentage of correct responses from the before-training phase to the final training session, indicating successful learning of the location of the sucrose-containing bottle (females: WT ctrl: BT vs. T5, *p* = 0.001; WT MIA: *p* = 0.0005; KO ctrl: *p* = 0.0056; KO MIA: *p* < 0.0001; males: WT ctrl - BT vs. T5: *p* = 0.0016, WT MIA - BT vs. T5: *p* < 0.0001, KO ctrl - BT vs. T5: *p* = 0.0334, KO MIA - BT vs. T5: *p* = 0.0430, significance levels are not shown in the figure). Neither the MIA nor the *Lcn2* gene deletion affected appetitively motivated learning in either males or females (Fig. 5D, E), except on training day T3 (T3 - WT ctrl vs. WT MIA: *p* = 0.0211; Fig. 5D). Additionally, KO females in the control group were less likely to initiate nose pokes toward the bottle that was later replaced with the sucrose solution (BT - WT ctrl vs. KO ctrl: *p* = 0.0378).

To confirm that a sucrose solution served as an appetitive stimulus for the tested mice, we assessed sweet water preference, calculated as the percentage of sucrose solution consumed, measured by lick count, relative to total fluid intake during each session. In females, no significant differences in sucrose consumption between experimental groups on any training day were found (Supplementary Fig. 5A). However, all female groups demonstrated a significant increase in sucrose intake compared to the before training phase, when only plain water was available (WT ctrl - BT vs. T5: *p* = 0.0003; WT MIA - BT vs. T5: *p* = 0.0009; KO ctrl - BT vs. T5: *p* = 0.0005; KO MIA - BT vs. T5: *p* < 0.0001; significance levels not marked in the figure).

Similarly, in males, no significant differences between groups on most training days were detected, except day 4, when KO MIA males consumed less sucrose solution than their WT MIA counterparts (T4 - WT MIA vs. KO MIA: *p* = 0.0435; Supplementary Fig. 5B). As observed in females, all-male groups exhibited significantly higher intake from the sucrose-containing bottle compared to the before training period with plain water (WT ctrl - BT vs. T5: *p* < 0.0001; WT MIA - BT vs. T5: *p* < 0.0001; KO ctrl - BT vs. T5: *p* = 0.049; KO MIA - BT vs. T5: *p* = 0.0379; significance levels not shown in the figure). Together, these results confirm that the sucrose solution was an appetitive stimulus for mice, as both sexes from all experimental groups consistently preferred the sweetened water.

### Lcn2 deficiency drive transcriptomic changes in brain development pathways similar to MIA

**Fig. 6:**
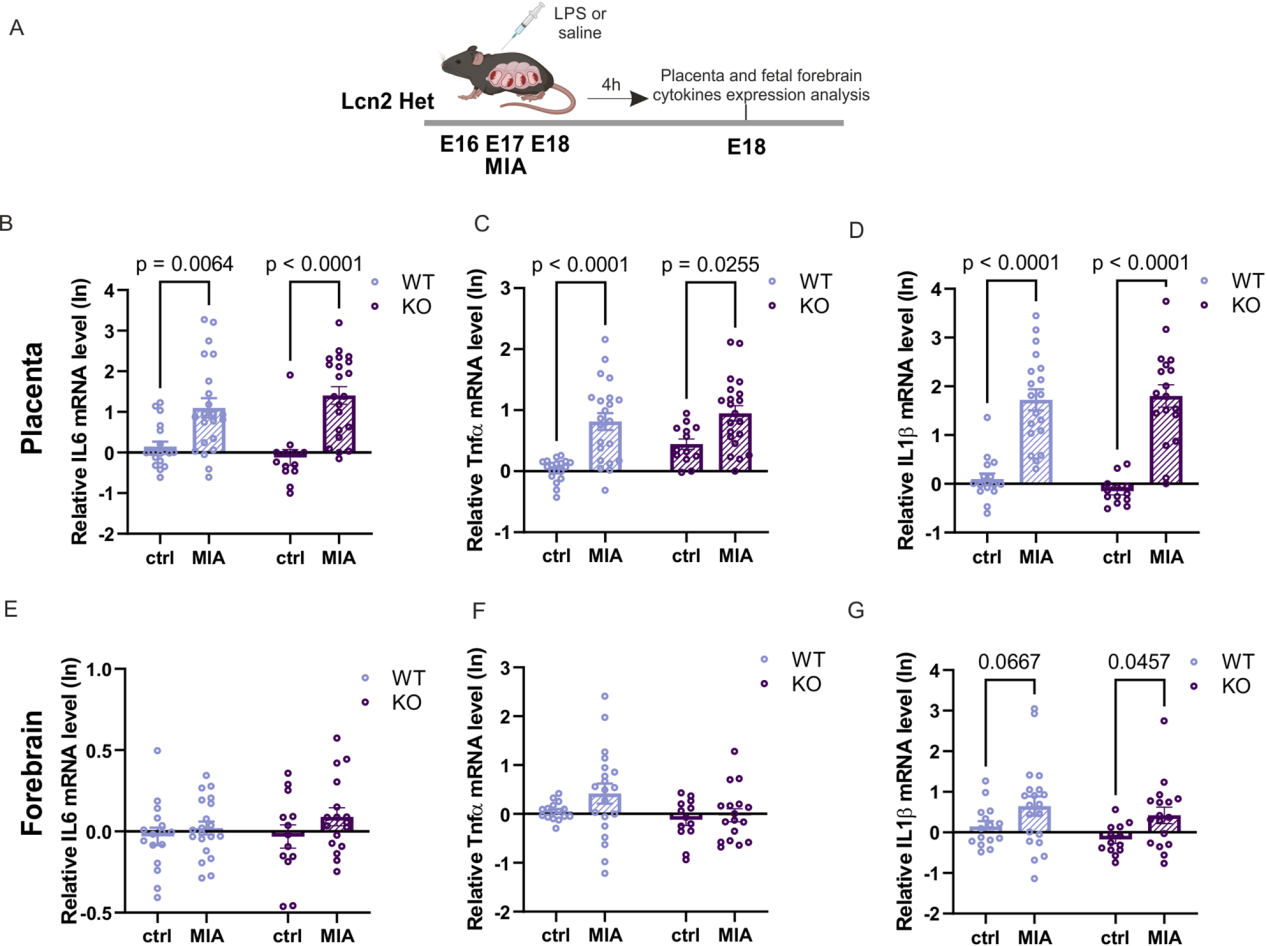
MIA induces proinflammatory cytokine expression in the placenta and fetal brain without detectable genotype-dependent differences. **A** Scheme of the experiment. LPS was administered to pregnant Lcn2 Het mice on embryonic days E16, E17, and E18. The placenta and fetal brain were collected 4 h after the final LPS injection. **B, C, D** mRNA expression of pro-inflammatory cytokines in the placenta. **B ***IL-6* mRNA level. Two-way ANOVA: procedure F (1, 71) = 34.13, *p* < 0.0001, genotype F (1, 71) = 0.009317, *p* = 0,9234, and interaction: F (1, 71) = 1,837, *p* = 0.1796. WT ctrl *n* = 19, WT MIA *n* = 22, KO ctrl *n* = 13, KO MIA *n* = 21. **C ***TNF-α* mRNA level: Two-way ANOVA: procedure F (1, 71) = 30.76, *p* < 0.0001, genotype F (1, 71) = 5.61, *p* = 0.0205, and interaction: F (1, 71) = 1,483, *p* = 0.2274. WT ctrl *n* = 19, WT MIA *n* = 22, KO ctrl *n* = 13, KO MIA n = 21. **D ***Il-1β* mRNA level. Two-way ANOVA: procedure F (1, 71) = 85.61, *p* < 0.0001, genotype F (1, 71) = 0.1380, *p* = 0.7114, and interaction: F (1, 71) = 0.5555, *p* = 0.4585. WT ctrl *n* = 19, WT MIA *n* = 22, KO ctrl *n* = 13, KO MIA n = 21. **E**, **F**, **G** mRNA expression of pro-inflammatory cytokines in fetal forebrain. **E ***IL-6* mRNA level. Two-way ANOVA: procedure F (1, 62) = 2.529, p = 0.1168, genotype F (1, 62) = 0.3958, *p* = 0.5316, and interaction F (1, 62) = 0.4106, *p* = 0.5240. WT ctrl *n* = 16, WT MIA *n* = 20, KO ctrl *n* = 13, KO MIA *n* = 17. **F ***TNF-α* mRNA level. Two-way ANOVA: procedure F (1, 62) = 2.203, *p* = 0.1428, genotype F (1, 62) = 4.056, *p* = 0.0484, and interaction F (1, 62) = 0.7794, *p* = 0.3807. WT ctrl *n* = 16, WT MIA *n* = 20, KO ctrl *n* = 13, KO MIA *n* = 17. **G ***Il-1β* mRNA level. Two-way ANOVA: procedure F (1, 61) = 7.649, *p* = 0.0075, genotype F (1, 61) = 1.920, *p* = 0.1709, and interaction F (1, 61) = 0.05133, *p* = 0.8215. WT ctrl *n* = 16, WT MIA *n* = 20, KO ctrl *n* = 13, KO MIA *n* = 17. On all graphs, the data were log-transformed (Y = ln(Y)). Data are presented as mean ± SEM, *n* = number of animals (males and females)

To explore the potential shared molecular mechanisms underlying maternal immune activation and Lcn2 knockout effects on brain function, we analyzed pro-inflammatory cytokine expression in the placenta and fetal forebrain following LPS-induced MIA in both WT and Lcn2 KO mice (Fig. 6). Maternal immune activation significantly altered *IL-6* (WT ctrl vs. WT MIA: *p* = 0.0064; KO ctrl vs. KO MIA: *p* < 0.0001), *TNF-α* (WT ctrl vs. WT MIA: *p* < 0.0001; KO ctrl vs. KO MIA: *p* = 0.0255), and *IL-1β* (WT ctrl vs. WT MIA: *p* < 0.0001; KO ctrl vs. KO MIA: *p* < 0.0001) expression in the placenta of both genotypes. No significant differences were observed between the WT MIA and Lcn2 KO MIA groups, suggesting broadly comparable placental inflammatory responses under these conditions. In contrast, inflammatory responses in the fetal brain were more limited, with *IL-1β* mRNA (WT ctrl vs. WT MIA: *p* = 0.0667; KO ctrl vs. KO MIA: *p* = 0.0457; Fig. 6G) being the primary cytokine showing alteration following LPS exposure. While IL-1β expression showed a trend toward an increase in the WT MIA group, this effect did not reach statistical significance. No statistically significant genotype-dependent differences were detected in any of the measured inflammatory markers.

**Fig. 7:**
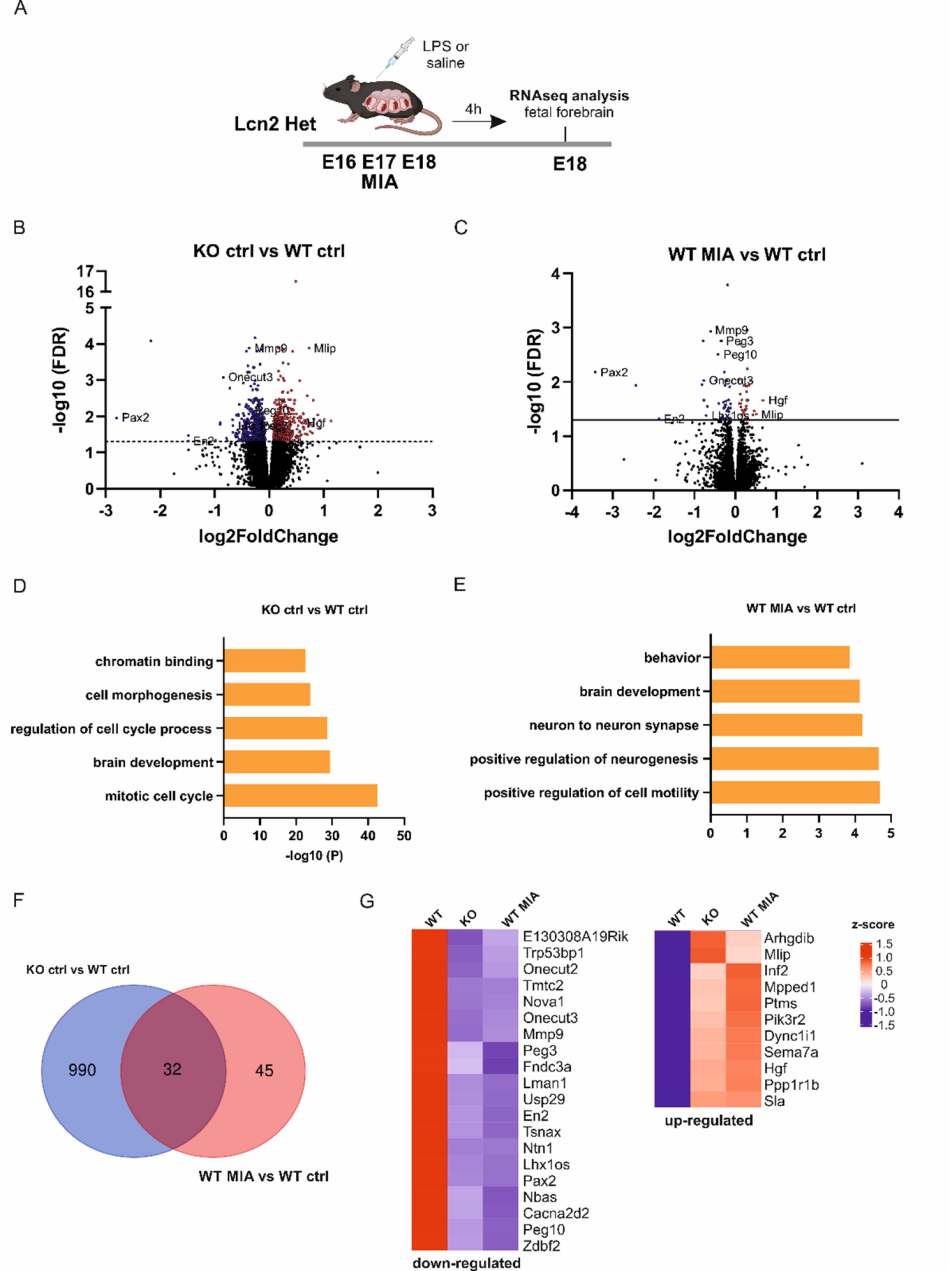
Convergent transcriptional signatures in Lcn2-knockout and MIA model. **A** Experimental timeline. Pregnant *Lcn2 *Het mice were injected with LPS or saline at E16, E17, and E18. 4 h after the last injection, the fetal forebrain was dissected for RNA-seq analysis. **B**,** C **A volcano plot illustrating DEGs in the forebrain of E18. The red dots represent significantly upregulated genes, the blue dots represent significantly downregulated genes (|log2 FC| ≥ 1 and FDR < 0.05), and the black dots represent insignificant differentially expressed genes. **B** DEGs in the forebrain of Lcn2 KO animals at E18. **C** DEGs in the forebrain of WT MIA at E18. **D**,** E **Gene ontology enrichment analysis using Metascape based on the genes deregulated (both up- and downregulated). A bar graph showing the top 5 clusters with the highest p-value. **F** A Venn diagram of unique and overlapping gene expression between Lcn2 KO and MIA groups. **G** The heat map of overlapping genes between Lcn2 KO and WT MIA. WT ctrl *n* = 6 (3 males, 3 females), KO ctrl *n* = 6 (3 males, 3 females), WT MIA *n* = 6 (3 males, 3 females)

To obtain an unbiased characterization of the transcriptional program induced by MIA, we performed bulk RNA sequencing (RNA-seq) four hours after the last LPS injection, using forebrain tissue from fetuses on gestational day E18. Data from both sexes were combined, as no major sex-specific differences were detected in MIA- or Lcn2 knockout-associated behavioral phenotypes. To define statistically significant changes in gene expression between MIA and control offspring, as well as WT and KO animals, a false discovery rate (FDR) threshold of < 0.05 was applied. Using the |log₂ fold change criteria| >1 and FDR < 0.05, we observed widespread transcriptional alterations in Lcn2 KO animals (Fig. 7B), with 573 genes downregulated and 449 upregulated. In the forebrains of offspring from LPS-treated dams, 46 genes were downregulated, and 31 were upregulated (Fig. 7C). Among these, *Lcn2* mRNA showed a 2.29-fold increase (*p* = 0.0356); however, this change did not remain statistically significant after correction for multiple comparisons. A complete list of upregulated and downregulated gene sets is provided in Supplementary Table 1.

The RNA-seq data were analyzed to identify differentially expressed genes (DEGs) between the groups and to assess the enrichment of Gene Ontology (GO) categories among these DEGs (Metascape). This analysis revealed significant enrichment in several GO categories, in DEGs in Lcn2 KO animals, and showed enrichment of ontologies associated with *brain development* (GO:0007420, 95/745 genes). In the DEGs in the MIA group, significant enrichment was also found in GO Biological Processes *brain development* (GO:0007420, 9/745 genes), *behavior* (GO:0007610, 8/634), and GO Cellular Components *neuron to neuron synapse* (GO:0098984, 7/414 genes; Supplementary Tables 2 and 3). Interestingly, stratification of RNA-seq data by sex did not substantially alter the gene ontology analysis, as similar biological themes related to brain or synapse development were affected in both males and females (Supplementary Fig. 6).

Next, we examined whether the transcriptomic changes exhibited shared expression patterns between MIA and Lcn2 KO animals. Although MIA induced relatively modest changes in gene expression, we found that 40% of the differentially expressed genes in the MIA group (31 out of 77) also showed altered expression in Lcn2 KO animals (Fig. 7G). Thirty-one shared genes represented a 6.9-fold enrichment over random expectation (representation factor = 6.9, *p* < 7.41 × 10⁻^19^; *p* values of hypergeometric probability significance test). Functional annotation of the overlapping genes was performed through manual analysis of published literature (PubMed search), focusing on neuronal relevance. Several genes within this set are known to be involved in brain development and have been implicated in neurodevelopmental disorders, suggesting that Lcn2 deficiency and MIA may converge on common molecular pathways essential for normal brain development (Table 1).

**Table 1:** Differentially expressed genes regulating brain development with a link to neurodevelopmental disorders in humans

GENE	Name	Function	Link with neurodevelopmental disorders	References
Pax2	Paired Box 2	transcription factor, present in the developing brain, regulates the differentiation of GABAergic neurons	autism spectrum disorder, intellectual disability, epilepsy	[54–56]
EN2	Engrailed homeobox 2	transcription factor, critical for brain development, regulates the number of GABAergic neurons	autism spectrum disorder	[57–62]
Hgf	Hepatocyte Growth Factor	growth factor, neurotrophic factor involved in synaptogenesis	autism spectrum disorder	[63–65]
Mmp9	Matrix metalloproteinase 9	metalloproteinase, regulates functional and structural plasticity	autism spectrum disorder, fragile X syndrome, schizophrenia	[66–72]
Sema7a	Semaphorin 7 A	is a key regulator of axon guidance, synapse formation	autism, developmental delay	[73, 74]
Ppp1r1b (DARPP-32)	Protein phosphatase 1 regulatory inhibitor subunit 1B	inhibitor of protein-phosphatase 1, plays a key role in neuronal signaling, particularly within dopamine pathways, regulates the number of GABAergic neurons	autism, schizophrenia	[75–77]
Cacna2d2	Alpha-2/delta-2 subunit of voltage-gated calcium channels	voltage-gated calcium channel, controls calcium-dependent signaling in neurons	epilepsy, schizophrenia	[78, 79]
Ntn1	Netrin-1	secreted protein, cue for axon guidance, promotes axonal branching, regulates the number and function of excitatory synapses	no direct link	[80–82]

## Discussion

This study strongly supports Lcn2 as a potent regulator of brain development. We demonstrate that Lcn2 is expressed in the brain during development and is upregulated following MIA, particularly in the hippocampus and neocortex. Behavioral analyses have revealed that Lcn2 deletion and MIA independently disrupt social behaviors and increase repetitive behaviors in offspring, without additive or synergistic effects, suggesting a potential occlusion of shared pathways. Notably, these behavioral alterations were selective, as cognitive performance in learning and memory tasks remained unaffected. Transcriptomic analysis of the fetal forebrain further supports this convergence, revealing a shared set of differentially expressed genes in both Lcn2-knockout and MIA-exposed fetal brains. These results suggest that even though increased Lcn2 expression following prenatal immune challenge does not appear to be the primary driver of behavioral disruptions in this model, Lcn2 likely plays an essential role in supporting neurodevelopment under physiological conditions.

While Lcn2 has been extensively studied in the context of adult brain pathologies, such as neuroinflammation and neurodegenerative processes [17, 19, 31, 36, 83, 84], its role during fetal brain development has remained largely unexplored. We demonstrate that Lcn2 expression in the mouse hippocampus is developmentally regulated, with mRNA levels detectable prenatally and peaking perinatally. Lcn2 expression has also been observed in the developing human brain [85] and in zebrafish embryos [24], indicating a potentially conserved role for this protein in neurodevelopment across species. Although no direct evidence for the role of Lcn2 in human neurodevelopmental disorders has been provided so far, it is important to note that either deletion or insertion identified within the 486.52–703.2 kb genomic region that encompasses the *Lcn2* gene in humans (according to the DECIPHER database; https://www.deciphergenomics.org/) has been found in patients with neurodevelopmental disorders’ symptoms including autism, global developmental delay, and intellectual disability.

Previous studies have shown that *Lcn2* expression increases in the adult mouse brain following systemic LPS administration [18, 19, 21, 22]. In the current study, we demonstrate that MIA induced by repeated low-dose LPS injections during late gestation leads to a robust upregulation of *Lcn2* mRNA in the fetal neocortex and hippocampus. This increase is accompanied by elevated maternal plasma Lcn2 levels, indicating a systemic inflammatory response associated with MIA. Importantly, the more sustained elevation of maternal Lcn2 relative to pro-inflammatory cytokines suggests that Lcn2 reflects a prolonged immune-activated state during MIA and may potentially serve as a biomarker of MIA-related immune activation.

Supporting the relevance of Lcn2 during early brain infection, a recent study reported elevated Lcn2 levels in both the blood and hippocampus of mice at postnatal day 4, 24 h after bacterial *Staphylococcus epidermidis* challenge [86], with immunofluorescence analyses indicating predominant localization of Lcn2 within brain endothelial cells. In the adult rodent brain, systemic inflammation similarly induces Lcn2 expression primarily in vascular endothelial cells, as well as in astrocytes and microglia [18–20]. Consistent with these observations, our data indicate that MIA-induced Lcn2 expression arises primarily from blood vessels. Since multi-target co-IF did not demonstrate the presence of Lcn2 in either neurons or astrocytes, we hypothesize that Lcn2 may act in a paracrine manner. Secreted Lcn2 is thought to interact with receptors such as 24p3R, MC4R, and LRP2 [24, 30, 87–93] on neurons, astrocytes, or microglia, thereby indirectly regulating their function.

Importantly, in our study, MIA-induced upregulation of Lcn2 was restricted to early developmental stages, with no detectable changes in hippocampal or cortical *Lcn2* mRNA expression in adult offspring of either sex. This finding suggests that Lcn2 may play a role primarily during acute immune-driven perturbations of brain development, rather than contributing to long-lasting inflammatory changes in the mature brain. In contrast, a previous RNA-sequencing study reported persistent, sex-specific transcriptional alterations in adult offspring following MIA, with significant changes observed in females but no detectable alterations in males [94]. Differences in experimental design, including the use of whole-brain homogenates versus region-specific analyses, as well as differences in the MIA paradigm (single high-dose versus repeated low-dose LPS exposure), may underlie these divergent observations.

Social behavior deficits are among the most consistently observed phenotypes in maternal immune activation models [95–101]. To assess the impact of MIA and *Lcn2* gene deletion on social behavior in mice, we used the Eco-HAB^®^ system, a validated tool for detecting social deficits in rodents [40, 41, 46, 50, 102]. It enables continuous tracking of spontaneous, long-term social interactions among group-housed animals in a semi-naturalistic environment, providing an ethologically relevant measure of sociability. Using this system, we have found that MIA disrupts sociability in wild-type offspring, as male and female mice born to LPS-treated dams were less willing to spend time with conspecifics than control mice. Interestingly, deletion of the *Lcn2* gene alone in control mice of both sexes produced a similar decrease in in-cohort sociability. Notably, Lcn2 deletion alone had a greater impact on social behavior in females than prenatal LPS exposure. Similar deficits in in-cohort sociability have been reported in various animal models of autism spectrum disorder, further supporting the relevance of the findings [40, 103].

Reduced interest in social stimuli, another marker of social dysfunction, was analyzed in the Eco-HAB^**®**^and modified three-chamber tests [102]. In both tests, MIA decreased social interest in WT females, while Lcn2 deletion reduced social preference only in males. In addition, the three-chamber test revealed an effect of LPS-induced reduction in interest toward the social stimulus in Lcn2 KO in females, which was not observed in Eco-HAB^®^. These discrepancies are probably due to differences in testing conditions, such as animals being assessed individually versus in a group, or variations in test duration. Nevertheless, these findings highlight that Lcn2 deficiency alone can impair preference for social stimuli, mimicking MIA-induced phenotypes. Similar reductions in social interest have been reported in offspring across various MIA models [101].

Although the social behavior experiments in Eco-HAB^®^ allowed us to characterize group behavior within genetically and treatment-homogeneous cohorts, they did not address the dynamics of interactions between mutant and control animals. Such cross-genotype housing can provide additional insight into the social modulation of behavioral phenotypes, as demonstrated in other automated systems, including IntelliCage, where co-housing with wild-type individuals partially restored cognitive functioning in mutant mice [104]. Incorporating this type of mixed-group design could increase the translational value of future studies, particularly in the context of understanding how social environments shape vulnerability and/or resilience. However, in the present work, such analyses were not feasible due to the large number of animals within a cohort required to perform Eco-HAB^®^ testing. Nevertheless, we consider mixed-cohort studies as an important potential direction for future research, especially for uncovering how social context may modulate phenotypic differences.

Besides impaired social interactions, repetitive behaviors are among the most consistently reported phenotypes following maternal immune activation [95, 97, 98, 105, 106]. Using the marble burying test, a well-established measure of repetitive behavior, we found that *Lcn2* knockout increased the number of marbles buried in both sexes. Interestingly, prenatal LPS exposure increased marble burying only in wild-type males, indicating a sex- and genotype-dependent effect. However, in Lcn2 KO males and females, MIA did not further alter this behavior. The absence of additive effects in KO mice exposed to MIA implies that Lcn2-dependent pathways may be a critical point of convergence in MIA-induced behavioral alterations. A similar phenomenon has been reported in Fmr1 KO mice, where Poly(I:C)-induced MIA did not exacerbate core autism-like behaviors [42].

Previous studies have reported inconclusive findings regarding the impact of MIA on cognitive function in offspring, with some reporting learning impairments following prenatal immune challenge, while others observed no significant deficits in learning and memory [107–110]. In our study, no significant effect of either MIA or *Lcn2* gene deletion on appetitively motivated spatial learning was found. Although learning deficits have previously been reported in Lcn2 KO mice [33, 34, 111], our Lcn2 knockout controls were also exposed to prenatal injection stress. This factor may have impacted brain development and contributed to phenotypic differences from previous studies. These findings suggest that Lcn2 and MIA may preferentially influence neural circuits underlying social and repetitive behaviors rather than broadly affect cognitive function.

The mechanisms by which prenatal infection disrupts neurodevelopment are still not fully understood. In this study, we compared gene expression profiles between MIA-exposed animals and Lcn2 knockout mice, which exhibit similar behavioral impairments. In both experimental groups, among differentially expressed genes (DEGs), gene ontology analysis showed enrichment in genes in the *brain development* category. These findings are in line with previous MIA transcriptomic studies identifying genes in specific categories related to central nervous system development [112–114]. We found a 6.9-fold enrichment of overlap genes between MIA and Lcn2 KO DEGs. On the molecular level, functional annotation of the shared genes indicated their role in control of inhibitory/excitatory balance and the regulation of functional and structural plasticity, highlighting their potential involvement in shaping neural circuit function (see Table 1). Our results are consistent with other studies showing that MIA may impact the GABAergic transmission by decreasing the number of GABAergic neurons [115–118]. Notably, animal models have also demonstrated that offspring exposed to maternal immune challenges exhibit synaptic dysfunction and reduced synaptic plasticity [119–121]. Moreover, mutations in many of the genes identified in our study are associated with increased susceptibility to autism spectrum disorder and other neurodevelopmental disorders in humans (Table 1). Interestingly, sex-based stratification did not substantially alter the outcomes of the gene ontology analysis, with similar biological themes observed in both males and females.

Despite the identification of shared transcriptional programs across our experimental groups, the signalling pathways underlying the convergence of these transcriptional changes remain unclear. Remarkably, even without an external inflammatory stimulus, Lcn2 deficiency may prime the fetal brain toward a transcriptional state characteristic of prenatal immune activation. However, the role of Lcn2 as an immunomodulatory protein remains controversial. While some studies have identified Lcn2 as a pro-inflammatory mediator that amplifies neuroinflammation [20, 24, 25, 28, 35, 122–124], other findings suggest a more complex role. For example, lack of Lcn2 has been associated with increased TNF-α and IL-6 expression and enhanced LPS-induced behaviors, suggesting a potential anti-inflammatory role [21]. However, other studies report no significant differences in brain inflammation between wild-type and Lcn2 knockout mice following LPS exposure [26, 29]. In line with these observations, MIA in our experimental paradigm led to increased placental expression of pro-inflammatory cytokines *(IL-6, TNF-α, IL-1β)* in both genotypes, without detectable differences between WT MIA and KO MIA groups. In the fetal brain, inflammatory changes were more restricted, with IL-1β emerging as the primary cytokine altered after LPS exposure, again without genotype-dependent effects. It should be noted, however, that inflammatory gene expression is highly dynamic, and analyses conducted at a single developmental time point may not capture transient, region-specific, or delayed genotype-dependent effects.

In the present study, both female and male offspring were included in order to capture potential sex-dependent effects of MIA. Although most of our results were broadly similar between males and females, some sex-related variation was observed. Sex-dependent effects have been reported in other MIA studies and are thought to reflect factors such as distinct neurodevelopmental trajectories and differential exposure to sex hormones, which can modulate vulnerability to prenatal immune challenges [125, 126]. However, the presence and magnitude of sex differences are highly variable: some models report greater male susceptibility, while others detect minimal differences, likely reflecting variation in the type of immune challenge, dosing, timing, and developmental stage assessed. Under the conditions examined here, MIA produced broadly similar effects in males and females, highlighting the importance of including both sexes in neurodevelopmental research.

While our findings provide novel molecular insights into the effects of MIA and Lcn2 on fetal brain development, they should be interpreted considering the limitations of the methods. Although bulk RNA sequencing offered valuable insights into shared molecular changes, single-cell transcriptomics and spatial transcriptomics in fetal forebrain tissues of Lcn2 KO and MIA mice would clarify the cellular origin of DEGs. Moreover, implementing inducible, cell-type-specific Lcn2 knockdown models following LPS exposure would help delineate the precise contribution of Lcn2 to LPS-induced behavioral outcomes. Another potential limitation of our study is the relatively modest number of genes differentially expressed following MIA. Although repeated LPS injections model maternal infection during key brain developmental stages, including neurogenesis and synaptogenesis [127, 128], this paradigm may also induce immune tolerance, dampening inflammatory and transcriptional responses [129, 130]. This phenomenon could partially explain the limited number of differentially expressed genes detected in the MIA condition.

## Conclusions

In conclusion, we have demonstrated that Lcn2 is a key factor in brain development, particularly in the formation of circuits that underlie social and repetitive behavior. The similar molecular and behavioral outcomes observed with Lcn2 deletion and MIA indicate that these interactions may converge on common developmental pathways.

## Supplementary Information

Supplementary Material 1. Supplementary Table 1.

Supplementary Material 2. Supplementary Table 2.

Supplementary Material 3. Supplementary Table 3.

Supplementary Material 4.

Supplementary Material 5. Supplementary Figures 1-6.

## Data Availability

The dataset supporting the conclusions of this article is available in the Gene Expression Omnibus (GEO) repository: [GSE303157].

## Abbreviations

Lcn2: Lipocalin-2
MIA: Maternal immune activation
NDDs: Neurodevelopmental disorders
ASD: Autism spectrum disorder
ADHD: Attention-deficit/hyperactivity disorder
LPS: Lipopolysaccharide
DEGs: Differentially expressed genes
