# Supplementary figures and images for: Lcn2 deficiency leads to long-lasting social impairments independent of maternal immune activation
Source: J Neuroinflammation. 2026 Feb 25;23:108. doi: 10.1186/s12974-026-03742-1 (PMC13040801; doi:10.1186/s12974-026-03742-1)

BLOTS for Figure 1 F, Pekala et al.,

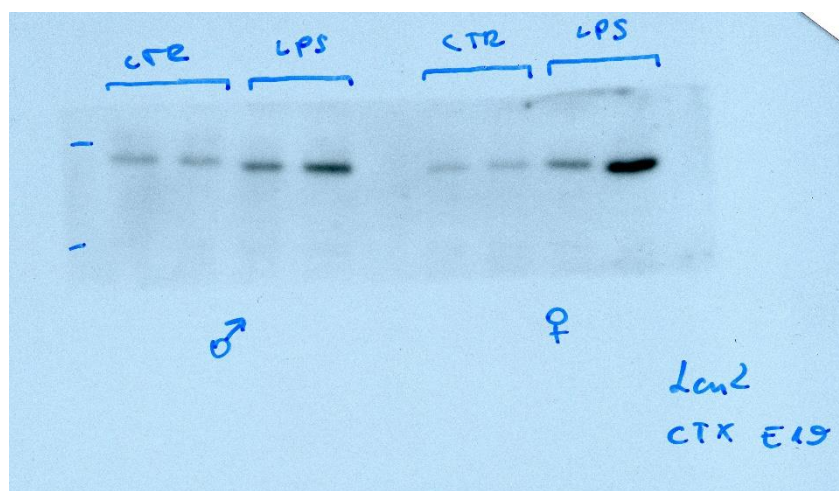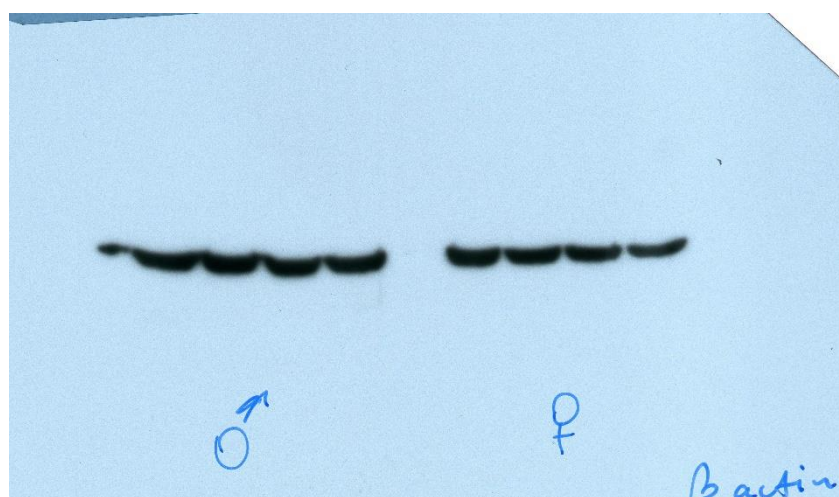

Supplement: Supplementary file 4 — Supplementary Material 4. [file 12974_2026_3742_MOESM4_ESM.pdf]
