# Supplementary material for: Lcn2 deficiency leads to long-lasting social impairments independent of maternal immune activation
Source: J Neuroinflammation. 2026 Feb 25;23:108. doi: 10.1186/s12974-026-03742-1 (PMC13040801; doi:10.1186/s12974-026-03742-1)
Supplement: Supplementary file 5 — Supplementary Material 5. Supplementary Figures 1-6. [file 12974_2026_3742_MOESM5_ESM.docx]

**Supplementary Figures:**


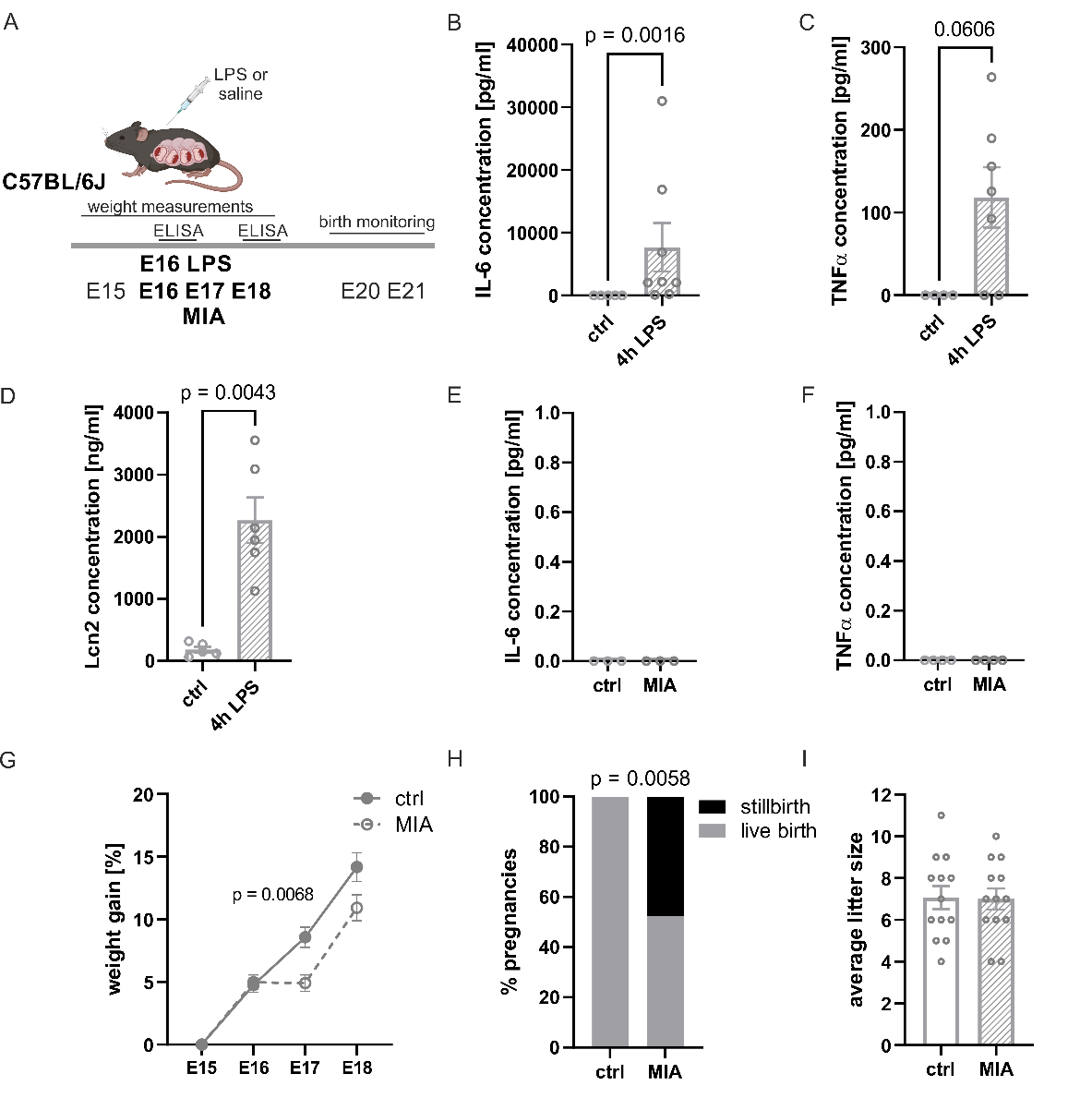


**Supplementary Fig. 1. MIA induces cytokine levels, affects weight gain in pregnant C57BL/6J females, and fetal survival.** **A.** Scheme of the experiment. LPS was administered to pregnant C57BL/6J mice on embryonic day E16, a single injection at 4 hours, or E16, E17, and E18 (MIA). Pregnant females' weight gain and offspring survival were analysed. **B.** IL-6 protein level in the plasma from pregnant dams at 4 hours after the LPS injection at E16, measured by ELISA. ctrl n = 4, 4 h LPS n = 8. Mann-Whitney test analysis. **C.** TNF-α protein level in the plasma from pregnant dams at 4 hours after the LPS injection at E16, measured by ELISA; ctrl n = 4, 4 h LPS n = 7. Mann-Whitney test analysis. **D.** Lcn2 protein level in the plasma from pregnant dams at 4 hours after the LPS injection at E16, measured by ELISA; ctrl n = 5, 4 h LPS n = 6. Mann-Whitney test analysis. **E.** IL-6 protein level in the plasma from pregnant dams at 4 hours after the last LPS injection at E18, measured by ELISA. ctrl n = 3, 4 h LPS n = 3. Mann-Whitney test analysis. **F.** TNF-α protein level in the plasma from pregnant dams at 4 hours after the last LPS injection at E18, measured by ELISA; ctrl n = 4, 4 h LPS n = 4. Mann-Whitney test analysis. **G**. Percentage of weight gain in pregnant females relative to their body weight on the day before the first injection. WT ctrl n = 11, WT MIA n = 12. Two-way repeated measures ANOVA: treatment: F (1, 20) = 4.939; p = 0.0379, time: F (1.437, 28.75 = 114.6; p < 0.0001, and interaction: F (2, 40) = 8.147; p = 0.0011, followed by Sidak’s post hoc test. Data are presented as mean ± SEM. **H.** Fetal survival, shown as the percentage of pregnancies resulting in live or stillbirths. WT ctrl n = 11, WT MIA n = 21. χ² test analysis. **I.** Average litter size born by C57BL/6J pregnant females after LPS or saline administration. ctrl n = 13, MIA n = 13, Student’s *t*-test. Data are presented as mean ± SEM, n = number of animals.

**
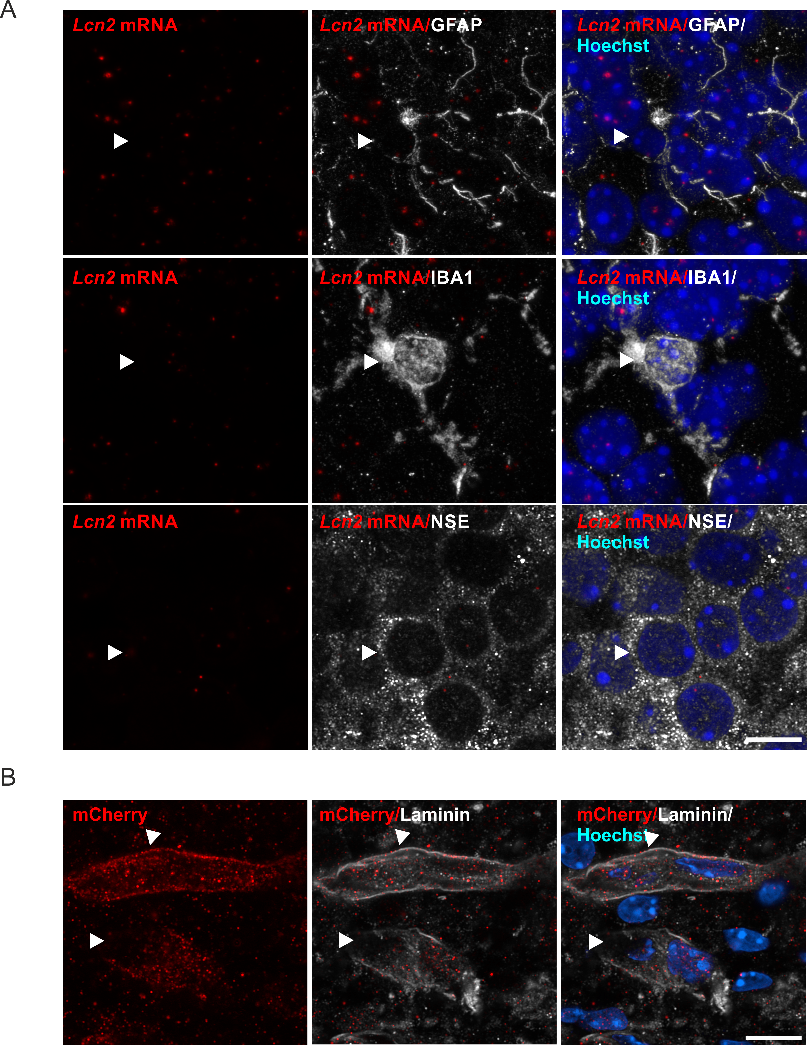
**

**Supplementary Fig. 2. Lcn is localized to laminin-positive blood vessels in the fetal brain. A.** RNAscope analysis of *Lcn2* mRNA in GFAP-positive astrocytes, Iba1-positive microglia, and NSE-positive neurons 24 hours after maternal immune activation (MIA). No *Lcn2* mRNA was detected in these cells. **B**. Immunofluorescent images of mCherry and Laminin in the cortex of Ngal-Luc2/mC mouse after MIA. Scale bar: 10 μm.


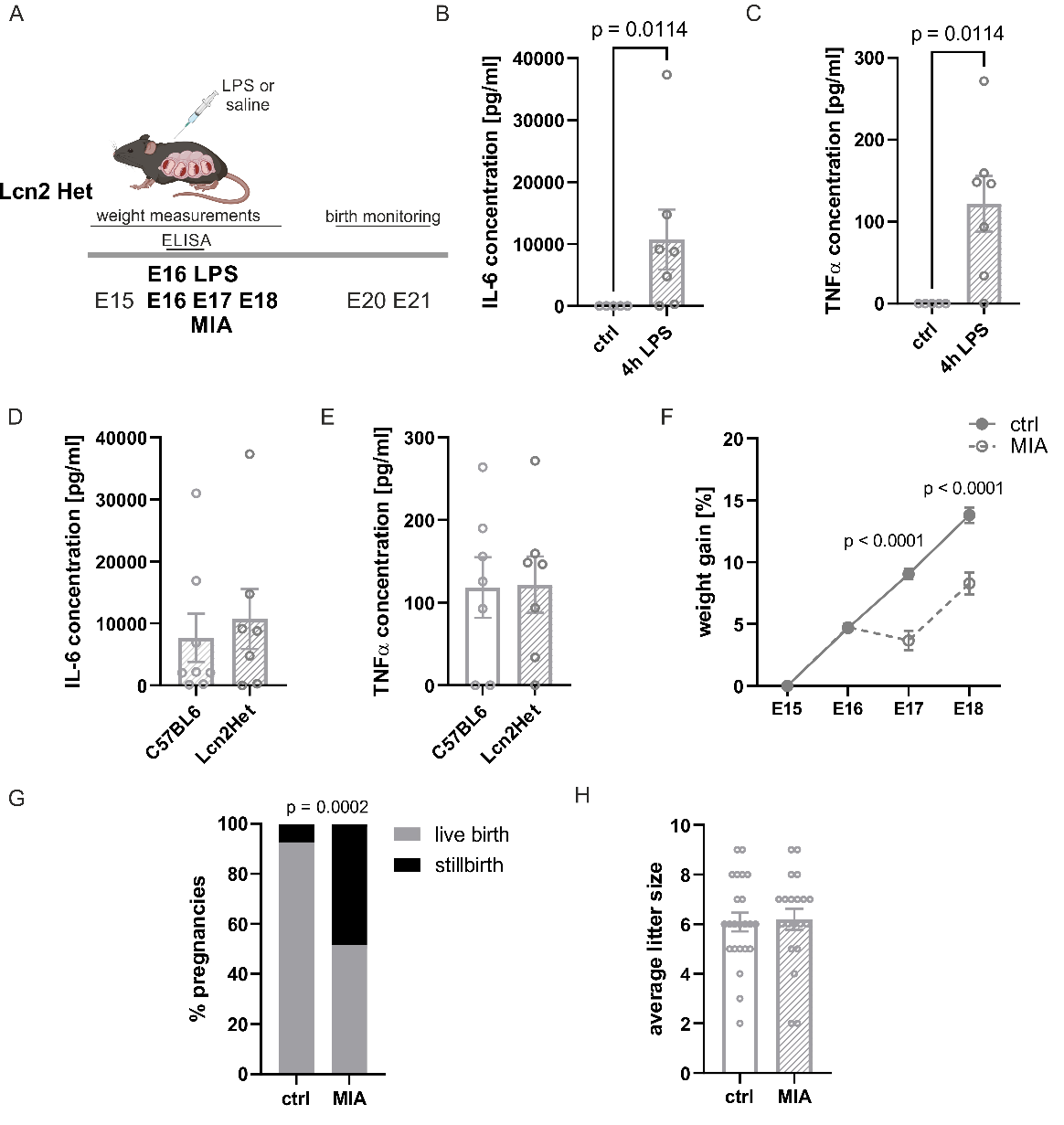


**Supplementary Fig. 3. Effect of MIA on weight gain in pregnant Lcn2 Het females and fetal survival.** **A.** Scheme of the experiment. LPS was administered to pregnant Lcn2 Het mice on embryonic days E16, a single injection at 4 hours, or E16, E17, and E18 (MIA). Pregnant females' weight gain and offspring survival were analyzed. **B.** IL-6 protein level in the plasma from Lcn2 Het pregnant dams at 4 hours after the LPS injection at E16, measured by ELISA: ctrl n = 5, 4h LPS n = 7; Mann-Whitney test analysis. **C.** TNF-α protein level in the plasma from Lcn2 Het pregnant dams at 4 hours after the LPS injection at E16, measured by ELISA: ctrl n = 5, 4h LPS n = 7; Mann-Whitney test analysis. **D.** IL-6 protein level in plasma from **C57BL/6J (data from S1.B) and** Lcn2Het at 4 hours after the LPS injection at E16, **C57BL/6J** n = 8, Lcn2Het n = 7. Mann-Whitney test analysis. **E.** TNF-α protein level in plasma from **C57BL/6J (data from S1.C) and** Lcn2Het at 4 hours after the LPS injection at E16, **C57BL/6J** n = 7, Lcn2Het n = 7. Mann-Whitney test analysis. **F.** Percentage of weight gain in pregnant females relative to their body weight on the day before the first injection. Ctrl n = 28, MIA = 34. Two-way repeated measures ANOVA: treatment: F (1, 60) = 26.89; p < 0.0001, time: F (1.633, 97.97) = 104.3; p < 0,0001, and interaction: F (2, 120) = 24.39; p < 0.0001, followed by Sidak’s post hoc test. Data are presented as mean ± SEM. **G.** Fetal survival is shown as the percentage of Lcn2 KO pregnancies resulting in live or stillbirths. WT ctrl n = 27, WT MIA n = 66. χ² test analysis. **H.** Average litter size born by Lcn2 Het pregnant females after LPS or saline administration. ctrl n = 23, MIA n = 20, Student’s *t*-test. Data are presented as mean ± SEM, n = number of animals.

**
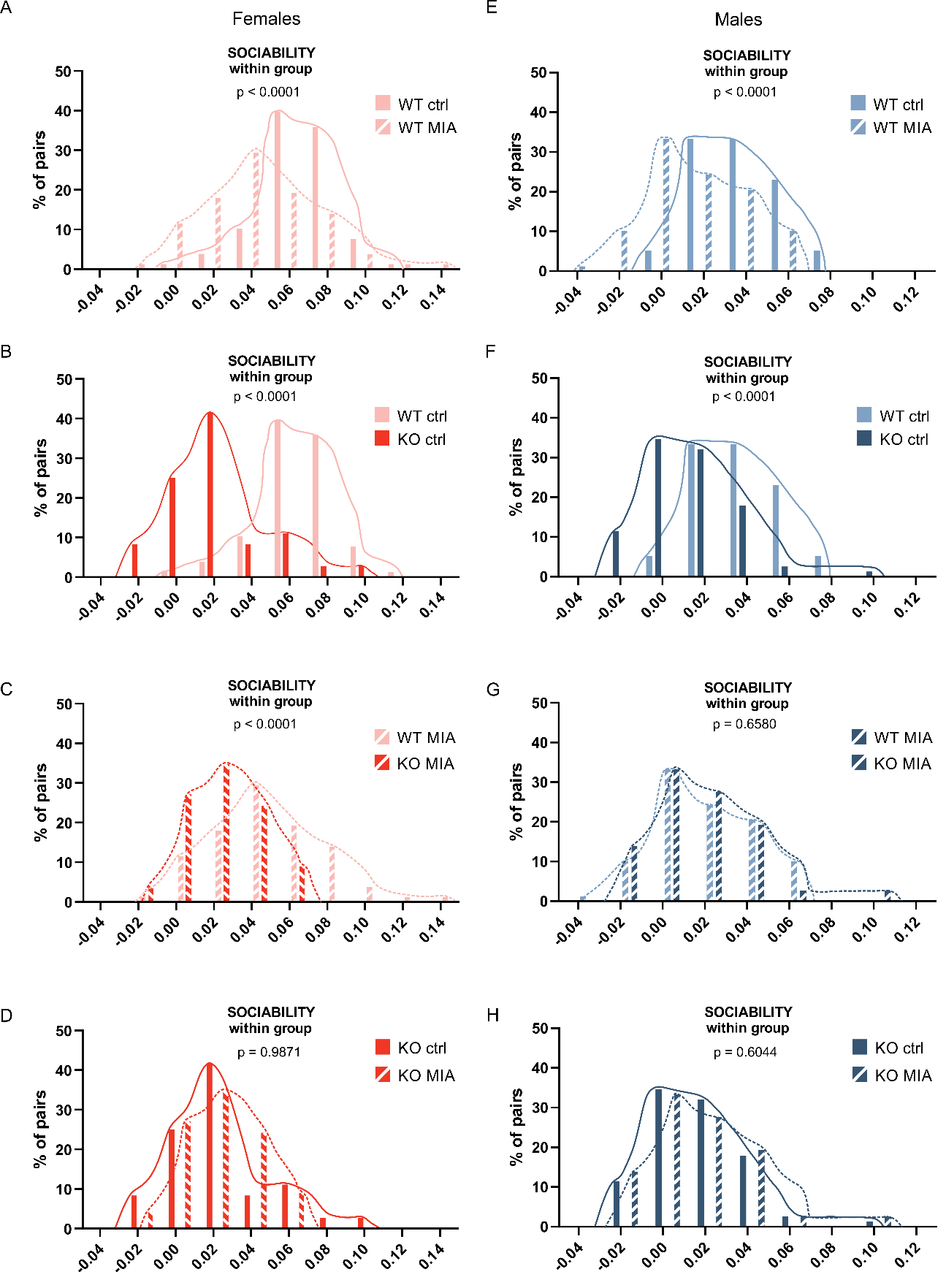
Supplementary Fig. 4**. **MIA or Lcn2 deletion alters sociability, represented by voluntary time spent together in pairs of mice**. The histogram illustrates the distribution of in-cohort sociability for all pairs of tested animal groups. **(A, B, C, D)** females, (**E, F, G, H**) males. Females: WT ctrl n = 13, WT MIA n = 13, KO ctrl n = 9, KO MIA n = 12; males: WT ctrl n = 12, WT MIA n = 13, KO ctrl n = 13, KO MIA n = 9. Kolmogorov–Smirnov test. Data are presented as a relative frequency distribution.

**
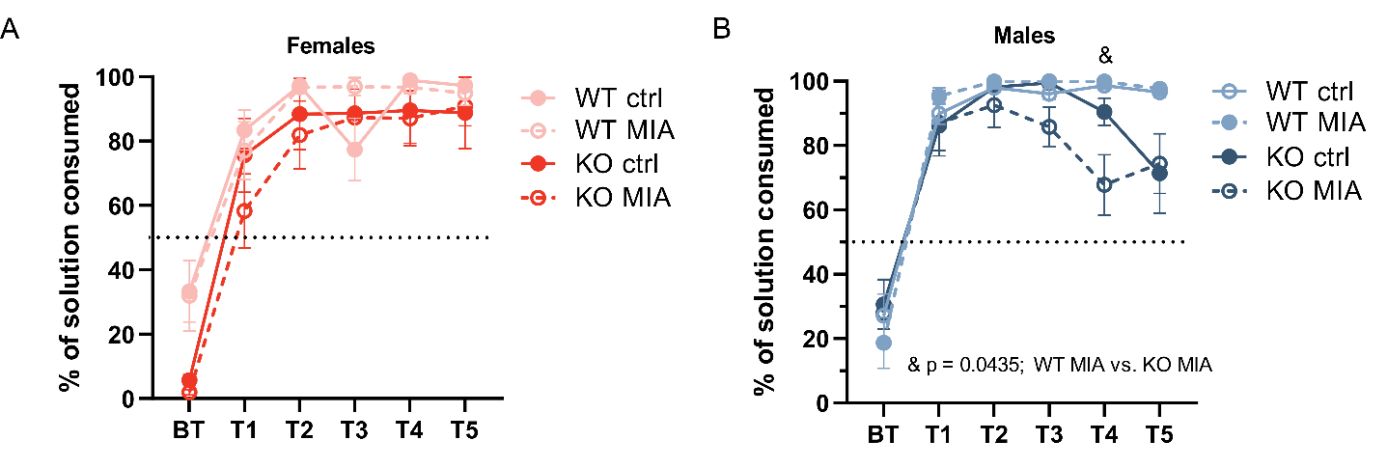
**

**Supplementary Fig. 5. Maternal immune activation or Lcn2 deletion does not influence sucrose consumption.** Sucrose consumption in females **(A)** and males **(B)** in IntelliCage. Preference for sweetened as the percentage of sugar solution consumed (measured by the number of licks) relative to the total fluid intake during a given session. **(A)** Three-way ANOVA: session F (2.198, 92.32) = 104.2; p < 0.0001, procedure F (1, 42) = 0.1118; p = 0.7397, genotype F (1, 42) = 4.195; p = 0.0468, session x procedure F (5, 210) = 1.378; p = 0.2337, session x genotype F (5, 210) = 2.955; p = 0.0134, procedure x genotype F (1, 42) = 0.2878; p = 0.5945, session x procedure x genotype F (5, 210) = 0.6052; p = 0.6960, followed by Tukey's post hoc test. Data are presented as mean ± SEM. **(B)** Three-way ANOVA: session: F (2.297, 94.18) = 104.0; p < 0.0001, procedure: F (1, 41) = 0.8240; p = 0.3693; genotype: F (1, 41) = 7.366; p = 0.0097, session x procedure F (5, 205) = 0.9636; p = 0.4412, session x genotype F (5, 205) = 4.624; p = 0.0005, procedure x genotype F (1, 41) = 1.377; p = 0.2474, session x procedure x genotype F (5, 205) = 1.163; p = 0.3288, followed by Tukey's post hoc test. Data are presented as mean ± SEM, n = number of animals.

**
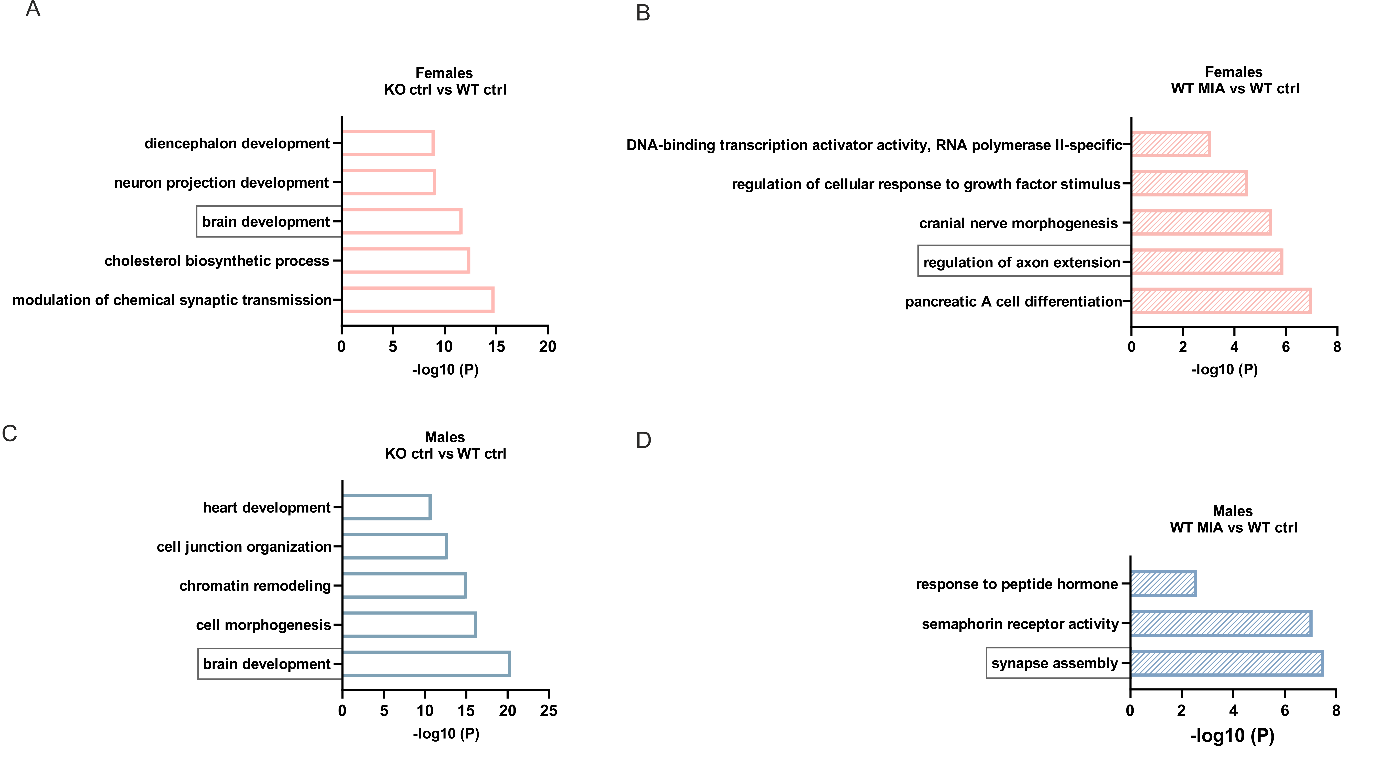
Supplementary Fig. 6. Gene ontology enrichment analysis using Metascape based on the genes deregulated (both up- and downregulated).** Bar graph showing the top 5 clusters with the highest p-value in females **(A)** and males **(C),** in the group of significantly changed genes: KO ctrl vs WT ctrl. Bar graph showing the top 5 clusters with the highest p-value in females **(B)** and 3 clusters in males **(D),** in the group of significantly changed genes in WT MIA vs WT ctrl.
